# Albumin Stimulates the Activity of the Human UDP-Glucuronosyltransferases 1A7, 1A8, 1A10, 2A1 and 2B15, but the Effects Are Enzyme and Substrate Dependent

**DOI:** 10.1371/journal.pone.0054767

**Published:** 2013-01-23

**Authors:** Nenad Manevski, Johanna Troberg, Paolo Svaluto-Moreolo, Klaudyna Dziedzic, Jari Yli-Kauhaluoma, Moshe Finel

**Affiliations:** 1 Centre for Drug Research, Faculty of Pharmacy, University of Helsinki, Helsinki, Finland; 2 Division of Pharmaceutical Chemistry, Faculty of Pharmacy, University of Helsinki, Helsinki, Finland; University Paris Diderot-Paris 7, France

## Abstract

Human UDP-glucuronosyltransferases (UGTs) are important enzymes in metabolic elimination of endo- and xenobiotics. It was recently shown that addition of fatty acid free bovine serum albumin (BSA) significantly enhances in vitro activities of UGTs, a limiting factor in in vitro–in vivo extrapolation. Nevertheless, since only few human UGT enzymes were tested for this phenomenon, we have now performed detailed enzyme kinetic analysis on the BSA effects in six previously untested UGTs, using 2–4 suitable substrates for each enzyme. We also examined some of the previously tested UGTs, but using additional substrates and a lower BSA concentration, only 0.1%. The latter concentration allows the use of important but more lipophilic substrates, such as estradiol and 17-epiestradiol. In five newly tested UGTs, 1A7, 1A8, 1A10, 2A1, and 2B15, the addition of BSA enhanced, to a different degree, the in vitro activity by either decreasing reaction’s *K*_m_, increasing its *V*_max_, or both. In contrast, the activities of UGT2B17, another previously untested enzyme, were almost unaffected. The results of the assays with the previously tested UGTs, 1A1, 1A6, 2B4, and 2B7, were similar to the published BSA only as far as the BSA effects on the reactions’ *K*_m_ are concerned. In the cases of *V*_max_ values, however, our results differ significantly from the previously published ones, at least with some of the substrates. Hence, the magnitude of the BSA effects appears to be substrate dependent, especially with respect to *V*_max_ increases. Additionally, the BSA effects may be UGT subfamily dependent since *K*_m_ decreases were observed in members of subfamilies 1A, 2A and 2B, whereas large *V*_max_ increases were only found in several UGT1A members. The results shed new light on the complexity of the BSA effects on the activity and enzyme kinetics of the human UGTs.

## Introduction

The human UDP-glucuronosyltransferase enzymes (UGTs) are located in the membrane of endoplasmic reticulum and play important roles in the metabolic elimination of lipophilic organic molecules of either external (xenobiotic) or internal origin [1], [2]. These enzymes transfer the glucuronic acid moiety from the cosubstrate, UDP-α-D-glucuronic acid (UDPGA), to nucleophilic groups on aglycone substrates that can vary considerably in chemical structure and physicochemical properties. Conjugation with glucuronic acid usually diminishes the pharmacodynamic activity of drugs and active drug metabolites, and also makes them more susceptible to excretion [3]. Since many drugs are subjected to glucuronidation, either directly or after phase I drug metabolizing reactions, the activity of UGTs may significantly affect their pharmacodynamic and pharmacokinetic profiles. As a result, improving methods for the prediction of UGT activity is an important research goal in drug development.

The main goal of in vitro glucuronidation assays is to accurately predict in vivo drug glucuronidation, both qualitatively and quantitatively [2]. Therefore, careful optimization of assay conditions is crucial for successful extrapolation of the in vitro results into valid conclusions about the in vivo UGTs activity. The inclusion of fatty acid free bovine serum albumin (abbreviated here as BSA even if not all BSA types are fatty acid free) was previously reported to significantly increase the in vitro activities of UGT1A9 and 2B7, regardless of whether recombinant enzymes or human liver microsomes (HLM) were used as an enzyme source [4]–[9].

Added BSA presumably binds lipophilic UGT inhibitors, such as fatty acids released from cells that are damaged during the preparation of HLM or recombinant UGT-enriched membranes [5]. The first studies on the BSA effects centered on UGT2B7, catalyzing zidovudine glucuronidation, and revealed that BSA inclusion largely lowers the reaction's *K*_m_ (provided substrate binding to BSA is taken into account), but with only a marginal effect on the reaction's *V*_max_, indicating that it increases the apparent substrate affinity [4], [5]. Similar effects of BSA inclusion were reported for 4-methylumbelliferone (4-MU) and propofol glucuronidation by UGT1A9 [6]. Subsequent studies from our laboratory demonstrated that while the BSA effects on UGT2B7 are reproducible, its effects on the activity of UGT1A9 are somewhat different since, in this case, it both lowers the *K*_m_ and increases the *V*_max_ values [8], [9]. The BSA effects on the activity of some other human UGTs were also tested, although not as thoroughly as with UGT2B7 and UGT1A9. It was reported that the BSA effects on UGT1A1 and UGT1A6 [6], [10], UGT1A4 [10], [11], and UGT2B4 [12] are generally minor.

The BSA effects are probably caused by the removal of lipophilic UGT inhibitors that were released during cells disruption. The differences in lipid composition between HLM, HEK293, or Sf9 insect cells may affect the manifestation of the BSA effects and might lead to differences in this respect between recombinant UGTs that were expressed in different systems, as well as between recombinant UGTs and the native ones in HLM. Based on the available experimental evidence, however, the differences in the BSA effects between HLM and recombinant UGTs that were expressed either in HEK293 cells [4]–[6], or Sf9 insect cells [8], [10], [12] are minor and most of the findings [4], [6], [8], [10], [12] suggest that, for a given UGT enzyme, the differences in lipid composition between HEK293 and Sf9 insect cells are not large enough to play a crucial role in the manifestation of BSA effects between different enzyme sources. Nevertheless, this does not exclude significant differences between different human UGT enzymes.

The possible major BSA impact on the in vitro activities of some UGTs and the variability in these effects among them prompted us to examine it in UGTs that have not been tested before. In addition, we tried to clarify, both in previously tested and the newly tested enzymes, whether or not the BSA effects are specific substrate and/or individual UGT dependent. Better and deeper understanding of the BSA effects on individual human UGTs will help us to improve the in vitro drug glucuronidation assays and, perhaps, to identify the site, or sites of inhibition. This, in turn, will teach us about the functions and mechanism of these complex enzymes.

We have now studied the BSA effects on ten different recombinant UGTs, six previously untested, namely 1A7, 1A8, 1A10, 2A1, 2B15, and 2B17, and four that were previously examined, UGTs 1A1, 1A6, 2B4 and 2B7, but mainly with different substrates and at a different BSA concentration. After careful optimization of assay conditions and measurement of substrates’ binding to BSA, we studied the enzyme kinetics of each of these recombinant UGTs in the absence and presence of BSA. The results of this extensive analysis are presented and discussed below.

## Materials and Methods

### Compounds and Reagents

4-Methylumbelliferone (4-MU; >99%, CAS 90-33-5), 1-naphthol (≥99%, CAS 90-15-3), 17α-estradiol (≥98%, CAS 57-91-0), 17β-estradiol (≥98%, CAS 50-28-2), UDP-α-D-glucuronic acid (UDPGA; ammonium salt, 98–100%, CAS 43195-60-4), 4-methylumbelliferone-β-D-glucuronide (≥98%, CAS 6160-80-1), 1-naphthol-β-D-glucuronide (>99%, CAS 83833-12-9), 17β-estradiol-17-β-D-glucuronide (≥98%, CAS 15087-02-2), 17β-estradiol-3-β-D-glucuronide (≥98%, CAS 14982-12-8), sodium phosphate monobasic dihydrate (≥99%, CAS 13472-35-0), and bovine serum albumin (BSA, ≥96%, CAS 9048-46-8, essentially fatty acid free, ≤0.01%) were purchased from Sigma-Aldrich (St. Louis, MO, USA). Entacapone (batch: 1044842) was a generous gift from Orion Pharma (Espoo, Finland). Entacapone-β-D-glucuronide was synthesized in our laboratory as described previously [13]. Magnesium chloride hexahydrate and perchloric acid were obtained from Merck (Darmstadt, Germany). Formic acid (98–100%) was from Riedel-deHaën (Seelze, Germany). Disodium hydrogen phosphate dihydrate was purchased from Fluka (Buchs, Switzerland). Dimethyl sulfoxide and solvents used for HPLC analyses were of liquid chromatography–mass spectrometry grade.

We have chemically synthesized 6-hydroxyindole by catalytic hydrogenation of 6-benzyloxyindole (>98.0%, CAS 15903-94-3, TCI Europe, Zwijndrecht, Belgium). The catalyst (Pd, 10% on activated carbon, 0.20 g, Sigma-Aldrich, St. Louis, MO, USA) was placed into two-necked 100 mL round-bottomed flask under inert atmosphere of argon. Methanol (20 mL) was then added, followed by a solution of 6-benzyloxyindole (0.30 g, 1.35 mmol) in methanol (20 mL). At this stage the argon atmosphere was replaced by dihydrogen and the reaction mixture was stirred at room temperature. The progress of the reaction was monitored by thin-layer chromatography on silica gel 60 F_254_ plates (Merck, Darmstadt, Germany) with *n*-hexane/ethyl acetate = 2∶1 eluent and UV detection at 254 nm (*R*_f_ = 0.3). After 2 h the reaction mixture was filtrated using the sinter funnel and Celite® 545 filtrating agent (CAS 68855-54-9, Sigma-Aldrich, St. Louis, MO, USA). The crude product was purified by Biotage high-performance flash chromatography Sp^1^ system (Uppsala, Sweden) using a 0.1 mm pathlength flow cell UV detector (fixed wavelength at 254 nm), Biotage SNAP 10 g flash cartridge, and *n*-hexane/ethyl acetate = 2∶1 mobile phase. The appropriate fractions were combined, and solvents were evaporated to dryness *in vacuo* to give 6-hydroxyindole (0.11 g, 0.83 mmol) as pale orange solids in 61% yield. The ^1^H NMR and ^13^C NMR spectra were recorded on a Varian Mercury 300 MHz (Varian, Palo Alto, CA, USA) as solutions in [D_6_]DMSO (Sigma-Aldrich, St. Louis, MO, USA). Multiplicities are indicated by s (singlet), d (doublet), dd (doublet of doublets), and m (multiplet). Chemical shifts (*δ*) are given in ppm relative to the residual DMSO signal: 2.50 and 39.52 ppm for ^1^H and ^13^C NMR, respectively. ^1^H NMR (300 MHz), *δ* (ppm): 10.63 (s, 1H), 8.82 (s, 1H), 7.28 (d, 1H, *J* = 8.4 Hz), 7.08 (dd, 1H, *J* = 3.3, 2.7 Hz), 6.74 (d, 1H, *J* = 2.4 Hz), 6.51 (dd, 1H, *J* = 8.4, 2.1 Hz), 6.26–6.24 (m, 1H). ^13^C NMR (75 MHz), *δ* (ppm): 152.8, 136.9, 123.1, 120.9, 120.2, 109.4, 100.8, and 96.4. The gas chromatograph–mass spectrometry (GC–MS) system consisted of the 5890A gas chromatograph and a 5970 mass selective detector (Hewlett Packard, Palo Alto, CA, USA) equipped with HP5–MS 12 m×0.25 mm column (Agilent Technologies, Palo Alto, CA, USA); GC–MS: *m/z* 133 [*M*]^+^, 104, 77, 51; *R*_t_ = 10.0 min. The HPLC-UV analysis was performed with Agilent 1100 HPLC instrument, Poroshell 120 EC-C18 column (4.6×100 mm, 2.7 µm; Agilent Technologies, Palo Alto, CA, USA), and UV detection at 254 nm (see *Analytical methods* below). Melting point was recorded on IA9100 digital melting point apparatus (Electrothermal Engineering Ltd., Essex, UK); mp = 124–129°C. The 6-hydroxyindole purity was assessed as 99% by ^1^H NMR, GC–MS, and HPLC-UV analyses.

### Enzyme Sources

Recombinant human UGTs 1A1, 1A6, 1A7, 1A8, 1A10, 2A1, 2B4, 2B7 and 2B17 were expressed as His-tagged proteins in baculovirus-infected insect cells as described previously [14]. Control samples, insect cell membranes without any human UGT, were prepared in the similar way by infecting the insect cells with baculovirus that does not encode any human UGT. Recombinant human UGT2B15 was purchased from BD Biosciences (Woburn, MA, USA; lot. 69457). Protein concentrations were determined by the BCA method (Pierce Biotechnology, Rockford, IL, USA).

### Drug Binding Assays

We measured the binding of 17α-estradiol, 17β-estradiol, and 6-hydroxyindole to 0.1% BSA and control insect cell membranes by rapid equilibrium dialysis (RED; Thermo Scientific, Rockford, IL, USA) as previously described [8]. The binding assays with 17α- and 17β-estradiol contained 1% DMSO in both sample and buffer chambers. The equilibration time was 8 h for the estradiols and 6 h for the 6-hydroxyindole binding assay. Nonspecific binding of the two estradiol isomers to the RED devices was rather high, nearly 40%. Nevertheless, due to the concentration independent nature of the estradiols binding to 0.1% BSA (see below), the non-specific binding to the RED device did not significantly affect the final result. The nonspecific binding of 6-hydroxyindole to the RED device was negligible (≤10%). The concentration range for the 17α-estradiol and 17β-estradiol was 2–100 µM, whereas for 6-hydroxyindole it was 5–750 µM. The unbound fraction of the test compound, *f*_u_, was calculated as follows:
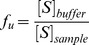
where [*S*]*_buffer_* and [*S*]*_sample_* are concentrations of the test compounds in the corresponding chambers.

### Drug Glucuronidation Assays

Stock solutions of all substrates were prepared in methanol and diluted with methanol to the desired concentrations before use. Appropriate amounts of these dilutions were transferred into 1.5 mL centrifuge tubes and the solvent was evaporated *in vacuo* at ambient temperature. The solvent was evaporated to avoid the presence of organic solvents in the incubation mixture. The solid residues were dissolved in the reaction mixture containing phosphate buffer (50 mM, pH 7.4), MgCl_2_ (10 mM), different BSA concentration (0.0, 0.1, or 1%), and enzyme source (0.02–0.2 mg/mL of total sample protein, depending on the enzyme source), to a final volume of 100 µL. The reactions with the two estradiol isomers, as well as reactions with 4-MU and 1-naphthol where the substrate concentrations exceeded 1000 µM, were performed in the presence of 1% DMSO. Alamethicin was not added to the incubations since it has no significant effect on the glucuronidation activity of recombinant UGTs [15], [16].

The mixtures were first incubated for 20 min at 0°C, followed by 5 min at 37°C, and the reaction was then initiated by the addition of UDPGA to a final concentration of 5000 µM. The 20 min preincubation at 0°C was performed to facilitate the dissolution of substrates in the reaction mixture, as well as to keep general consistency with HLM and HIM assays that are treated in this way due to the use of alamethicin (even if HLM and HIM assays were not included in this work) [17], [18]. The 5 min preincubation at 37°C was performed to prewarm the reaction mixture to the assay temperature. The reactions were carried out at 37°C for 10–60 min, protected from light. Negative controls, including reactions without UDPGA or without the aglycone substrate, were carried out for each set of assays. The glucuronidation reactions were terminated either by the addition of 60 µL ice-cold 4 M perchloric acid/methanol (1∶5 mix) or, in the case of the estradiols glucuronidation assays, by the addition of 100 µL 5% acetic acid in methanol. Following reaction termination, the tubes were kept at –20°C for 30–60 min and then centrifuged at 16000 *g* for 5 min. Aliquots of the resulting supernatants were transferred to dark glass vials and subjected to HPLC or UPLC analyses.

The protein concentrations and incubation times for the enzyme kinetic assays were selected based on preliminary assays in order to ensure that the product formation was within the linear range with respect to both protein concentration and time, and that the substrate consumption during the reaction was less than 10%. The initial velocity measurements were performed in either duplicates or triplicates and they are presented as the average values and S.E.

The glucuronidation rates by recombinant enzymes are reported as “expression normalized” values. The relative expression level of each recombinant UGT that was produced in our laboratory was determined using immunoblotting, as previously described [14], [19]. The dot blot assays were performed in triplicate and the average value was used for normalization. The relative expression level of UGT1A10 was set to 1.00 and, in comparison to it, the relative expression levels of UGTs 1A1, 1A6, 1A7, 1A8, 2A1, 2B4, 2B7 and 2B17 were 1.43, 1.69, 23.02, 2.08, 2.79, 0.65, 7.00 and 2.67, respectively. The “expression normalized” glucuronidation rates were calculated by dividing the measured glucuronidation rates with relative expression level of the each given recombinant UGT. One of the reasons for carrying out rates normalization (or correction according to relative expression levels) was to avoid the very high (but not realistic when compared to other UGTs) glucuronidation rates by UGTs 1A7 and 2B7, the enzymes that had the highest relative expression levels in the batches that were used in this study, 23 and 7, respectively. The relative expression levels of the other UGT enzymes exhibited much less variability and their “normalized” glucuronidation rates were generally similar to the “measured” rates. Due to lack of appropriate His-tag, the expression level of the commercial recombinant UGT2B15 could not be determined and, consequently, the results with this UGT are presented as the measured glucuronidation rates.

### Analytical Methods

The HPLC system consisted of an Agilent 1100 series degasser, binary pump, 100-vial autosampler, thermostated column compartment, multiple wavelengths UV detector, and fluorescence detector (Agilent Technologies, Palo Alto, CA, USA). For all HPLC separations we have used Poroshell 120 EC-C18 column (4.6×100 mm, 2.7 µm; Agilent Technologies, Palo Alto, CA, USA), column temperature of 40°C, and eluent flow rate of 1 mL/min. The resulting chromatograms were analyzed with Agilent ChemStation software (rev. B.01.01) on Windows XP Professional. The specific details of used analytical methods are presented in Table 1. The UPLC system consisted of a Waters Acquity UPLC equipped with column manager, sample manager, binary solvent pump, and photodiode array UV detector (Waters, Milford, MA, USA). The UV detector was equipped with high-sensitivity 2.4 µL flow cell. For UPLC separations we have used Acquity UPLC BEH C18 (2.1×100 mm, 1.7 µm, Waters, Milford, MA) column equipped with a pre-column, column temperature of 40°C, and flow rate of 0.5 mL/min. The resulting chromatograms were analyzed with Empower 2 software (Build 2154, Waters, Milford, MA, USA) on Windows XP Professional operating system. All specific details of the analytical conditions are presented in Table 1. The limits of detection and quantification were estimated based on signal-to-noise ratios of 3 and 10, respectively.

**Table 1:** Analytical conditions in the separation and quantification of glucuronides.

Analyte	Instrument	Eluents and gradient	Injection volume	Detection parameters	Retention time	Quantification; LOD and LOQ^1^
			*µL*	*wavelength, nm*	*min*	*Standard curve; µM*
4-MU-β-D-glucuronide	HPLC	A: 0.1% Formic acid; B: Acetonitrile; 0–3 min, 20→50% B; 3–3.1 min, 50→20% B; 3.1–5 min, 20% B	5–40	Fluorescence, λ_ex_ 316, λ_em_ 382	3.33	Authentic standard; 0.001, 0.003
1-Naphthol-β-D-glucuronide	HPLC	A: 0.1% Formic acid; B: Acetonitrile; 0–3 min, 30→50% B; 3–4 min, 50% B; 4–4.1 min, 50→30% B; 4.1–6 min, 30% B	20	Fluorescence, λ_ex_ 282, λ_em_ 335	2.30	Authentic standard; 0.001, 0.003
Entacapone-β-D-glucuronide	UPLC	A: 50 mM Phosphate buffer, pH 3.0; B: Acetonitrile; 0–3 min, 20→30% B; 3–3.2 min, 30→80% B; 3.2–4 min, 80% B; 4–4.1 min, 80→20% B; 4.1–6 min, 20% B	20–40	UV, 309	2.18	Authentic standard; 0.052, 0.172
17β-Estradiol-3-β-D-glucuronide and 17β-Estradiol-17-β-D-glucuronide	HPLC	A: 50 mM Phosphate buffer, pH 3.0; B: Acetonitrile; 0–2 min, 25% B; 2–5 min, 25→50% B, 5–9 min, 50% B, 9–9.1 min, 50→25% B, 9.1–10 min, 25% B	20–50	UV, 225; Fluorescence, λ_ex_ 225, λ_em_ 312	4.66 and 5.26	3-glucuronide based on 17β-estradiol’s UV absorption^2^; 0.066, 0.221 (UV); 0.019, 0.065 (FLD); 17-glucuronide authentic standard; 0.007, 0.024
17α-Estradiol-3-β-D-glucuronide and 17α-Estradiol-17-β-D-glucuronide	HPLC	A: 50 mM Phosphate buffer, pH 3.0; B: Acetonitrile; 0–2 min, 25% B; 2–5 min, 25→50% B, 5–9 min, 50% B, 9–9.1 min, 50→25% B, 9.1–10 min, 25% B	20	Fluorescence λ_ex_ 230, λ_em_ 308	5.01 and 5.71	Based on 17α-estradiol’s UV absorption^2^; 0.078, 0.283 (UV); 0.017, 0.068 (FLD)
6-Hydroxyindole-β-D-glucuronide	HPLC	A: 0.1% Formic acid; B: Acetonitrile; 0–6 min, 10→50% B; 6–6.8 min, 50% B; 6.8–6.9 min, 50–10% B; 6.9–10 min, 10% B	20	UV, 268; Fluorescence, λ_ex_ 268, λ_em_ 350	4.02	Based on 6-hydroxyindole’s UV absorption^2^; 0.287, 0.955 (UV); 0.001, 0.003 (FLD)
17β-Estradiol	HPLC	A: 50 mM Phosphate buffer, pH 3.0; B: Acetonitrile; Isocratic, 2.5 min, 60% B	50	Fluorescence, λ_ex_ 216, λ_em_ 313	2.10	Authentic standard; 0.004, 0.014
17α-Estradiol	HPLC	A: 20% Acetonitrile; B: Acetonitrile; Isocratic. 2.5 min, 55% B	50	Fluorescence, λ_ex_ 268, λ_em_ 296	1.75	Authentic standard; 0.005, 0.016
6-Hydroxyindole	HPLC	A: 0.1% Formic acid; B: Acetonitrile; Isocratic, 3 min, 40% B	40	UV, 268	1.68	Authentic standard; 0.060, 0.201

1 LOD, limit of detection; LOQ, limit of quantification; values are calculated assuming maximal injection volume;

2 The UV signal for was correlated with fluoresence for enhanced sensitivity.

### Analysis of Enzyme Kinetics

The enzyme kinetic parameters were obtained by fitting kinetic models to the experimental data using GraphPad Prism version 5.04 for Windows (GraphPad Software Inc., San Diego, CA, USA). The best model was selected based on visual inspection of the Eadie-Hofstee plots, residuals graphs, parameter S.E. and 95% confidence intervals estimates (95% CI), the calculated *r* ^2^ values, and corrected Akaike’s information criterion. In assays containing BSA, the total concentrations of substrate were corrected according to the measured drug binding to BSA, under the specific conditions of each glucuronidation assay. The statistical significance of the difference in enzyme kinetics parameters upon BSA addition was tested with extra sum-of-squares F-test (included in the GraphPad software, [20]. The initial glucuronidation rates were fitted to the following equations:

### Michaelis-Menten Model


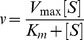
(1)

Where *v* is the initial velocity of the enzyme-catalyzed reaction, [S] is the substrate concentration, *V*_max_ is the limiting reaction velocity at saturating substrate concentrations, and *K*_m_ is the Michaelis-Menten constant (concentration of substrate at 0.5 of *V*_max_).

### Substrate Inhibition Model


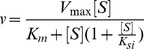
(2)

Where the *K*_si_ constant describes substrate inhibition in a mechanism in which a substrate molecule binds to an already formed enzyme-substrate complex, and thus acts as an uncompetitive inhibitor of the reaction.

### Sigmoidal Model (Hill Equation)


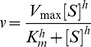
(3)

Where *S*_50_ is the substrate concentration at 0.5 of *V*_max_ (analogous to *K*_m_ in Michaelis-Menten model), and *h* is the Hill coefficient.

### Biphasic Model [21]


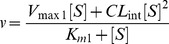
(4)

Where *V*_max1_ and *K*_m1_ are estimated from the curved portion of the plot at lower substrate concentrations. The CL_int_ represents the ratio of *K*_max2_/*K*_m2_ and describes the linear portion of the plot at higher concentrations of substrate.

For reactions that follow the Michaelis-Menten or substrate inhibition saturation profile, the intrinsic clearance was calculated as CL_int_ = *V*_max_/*K*_m_. For reactions exhibiting sigmoidal kinetics, however, we calculated the maximum clearance, CL_max_, an alternative parameter that expresses the clearance of fully activated enzyme before the saturation [22]:
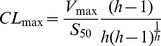
(5)


The relative *K*_m_, *V*_max_, CL_int_, and CL_max_ in the presence of BSA were calculated as 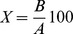, where B and A are the corresponding enzyme kinetic parameters in the presence and absence of BSA, respectively. The errors for *X* were estimated based on the general equation for the propagation of independent and random errors 
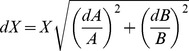
, where *X*, *A*, and *B* are the average values, and *dX*, *dA*, and *dB* are the corresponding errors [23]. The errors for CL_max_ were calculated according to the general equation for the propagation of uncertainty in a function of several variables 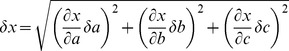, where *a* is *V*_max_, *b* is *S*_50_, *c* is *h*, and *δa*, *δb*, and *δc* are the corresponding errors [23].

## Results

**Figure 1:**
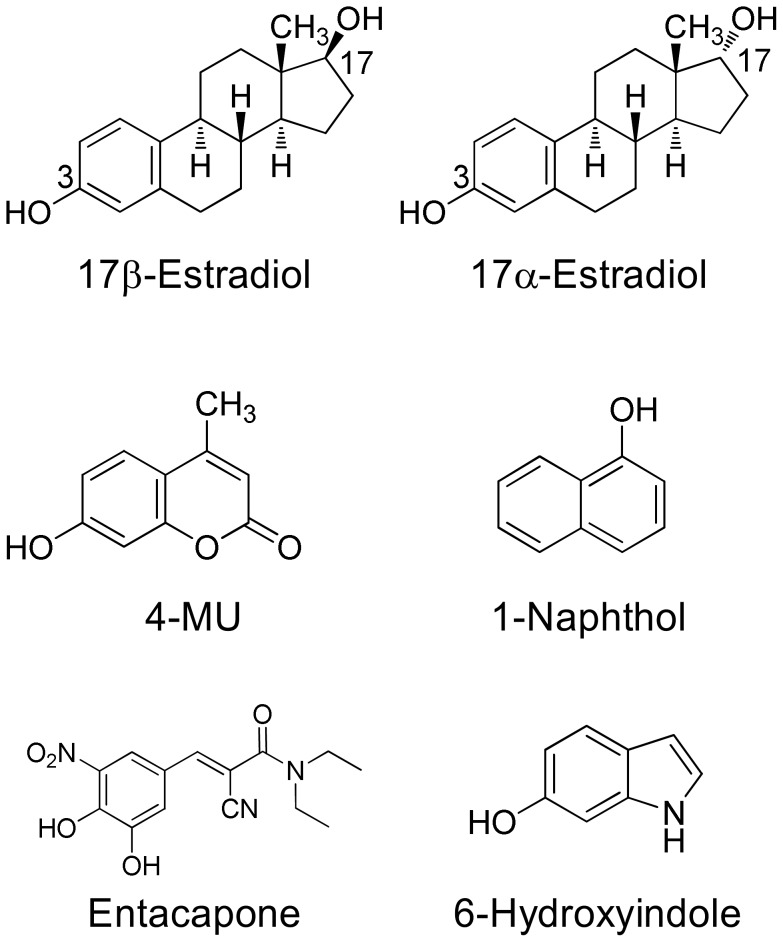
The chemical structures of the substrates that were used in this study. In 17α- and 17β-estradiol both the 3-OH and the 17-OH can be conjugated, mostly by different UGTs (see Figs. 2 and 3). In the case of entacapone, the glucuronidation occurs on hydroxy group in position 3.

In this study we have employed six different aglycone substrates to examine the BSA effects in ten different UGT enzymes. The substrates were 17α-estradiol (also called 17-epiestradiol), 17β-estradiol (the physiological estradiol), entacapone, 6-hydroxyindole, 1-naphthol, and 4-MU (Fig. 1). The desire to include the two estrogens in this study, due to their suitability for studies with many human UGTs [24] was one of the major reasons to use a lower BSA concentration than previously used in most studies, particularly since results from our laboratory demonstrated that most of the BSA effects are detectable in the presence of 0.1% and there is no real need to use 2% BSA in such assays [8]. Out of the ten UGT enzymes, 1A7, 1A8, 1A10, 2A1, 2B15, and 2B17 have not been tested for BSA effects before, while the remaining four, UGTs 1A1, 1A6, 2B4, and 2B7, were tested previously, mostly with other substrates [4]–[6], [10], [12] and partly serve as positive controls in this study.

### Binding of Substrates to 0.1% BSA and to Control Insect Cell Membranes

Added albumin in the UGT assays often binds aglycone substrates and decreases their actual concentration in solution. Therefore, in order to reach meaningful conclusions about the BSA effects on the dependence of glucuronidation rates on substrate concentration, it is necessary to accurately measure the unbound fraction of the substrate (*f*_u_ values) at the given BSA concentration. For three out of six selected substrates in this study, 1-naphthol, 4-MU and entacapone, we have previously determined the respective *f*_u_ values in the presence of either 0.1% or 1% BSA [8].

In this study, we measured the binding of 17α-estradiol, 17β-estradiol, and 6-hydroxyindole to 0.1% BSA and to control insect cell membranes by RED assays [8]. The binding of 17α- and 17β-estradiol to 0.1% BSA was independent of substrate concentration and yielded *f*_u_ values of 0.49±0.01 and 0.48±0.01 (average ± S.E.), respectively, indicating that the stereochemistry at carbon C-17 does not affect the binding of estradiols to albumin. The *f*_u_ values in the presence of 0.1% BSA (above) are much higher than the values in the presence of 0.5% and 2% BSA, namely *f*_u_ = 0.1 and 0.04, respectively [10], [25], an outcome that makes full kinetic analysis of these estradiols glucuronidation much easier. Nevertheless, it should be noted that, for solubility reasons, the *f*_u_ values of estradiols binding to 0.1% BSA were measured in the presence of 1% DMSO, the same concentration that was used in 17α- and 17β-estradiol glucuronidation assays. It is noteworthy that the presence of 1% DMSO in the reaction mixture does not reduce the estradiol glucuronidation activity of the human UGTs [15]. Nevertheless, this issue was re-examined here by testing the 1% DMSO influence on entacapone glucuronidation by UGT1A8, in both the absence and presence of 0.1% BSA. Entacapone was selected since its solubility was independent of presence or absence of DMSO (see below). It was found that addition of 1% DMSO resulted in only a minor reduction of the UGT activity (≤15%), and did not significantly affected the enzyme kinetic parameters or the stimulatory effects of 0.1% BSA addition.

The binding of 6-hydroxyindole to 0.1% BSA was generally low, but partly concentration dependent, yielding high *f*_u_ values, 0.84–0.97 at 5–750 µM substrate concentration. In order to estimate the *f*_u_ at any given concentration of 6-hydroxyindole, we fitted the experimental data points with empirical equation, and estimated the *f*_u_ from the interpolated function (data not shown).

The binding of 17α-estradiol, 17β-estradiol, and 6-hydroxyindole to 0.2 mg/mL of control insect cell membrane (total protein in the membrane), the highest protein concentration used in this work, was negligible.

### Experiments to Optimize the Assay Conditions of Enzyme Kinetic Analyses

Prior to enzyme kinetic assays, we performed preliminary tests to find the optimal experimental conditions for each analysis. The main focus of these experiments was on optimizing the BSA concentration, the protein concentration (total protein concentration in the UGT-enriched insect cells membranes), and possible need for DMSO addition (if clearly advantageous at high substrate concentration, it is best be included already with low concentrations of the given substrate). We included 1% DMSO in the assays with 17α- and 17β-estradiol, as well as in the assays with high concentrations of 4-MU and 1-naphthol (when [*S*] ≥1000 µM). Addition of 1% DMSO in these cases was necessary due to poor aqueous solubility of estradiols [26], as well as visually observed precipitation of substrates and poor reproducibility that we have observed in preliminary assays with high concentrations of 4-MU and 1-naphthol. DMSO addition had no significant effect on the solubility of entacapone and 6-hydoxyindole (results not shown) and, therefore, it was not included in assays using these two latter substrates.

In preliminary experiments with UGT1A7, UGT1A10 and UGT2A1, using 4-MU as a common substrate, enzyme kinetic assays were performed in the presence of either 0.1% or 1% BSA. The results of these experiments were in good agreement with the already published results with UGT1A9 [8], namely only a small increase of enzyme activity was achieved by replacing 0.1% with 1% BSA. Specifically, the addition of 1% BSA, compared to 0.1%, resulted in approximately 20–30% of further *K*_m_ decrease and 10–15% of increase in *V*_max_ values, depending on the enzyme source. These results are in good agreement with published data on 4-MU glucuronidation that were performed in the presence of both 0.1 and 1% BSA, using UGTs 2B7 and 1A9 that were expressed in HEK293 cells [5], [6]. Similar to our results, somewhat higher degree of the 4-MU *K*_m_ decrease, by about 20 to 30%, was observed when 1% of BSA was used instead of 0.1% BSA [5], [6]. The use of 1% BSA instead of 0.1% BSA, however, led to a more profound *K*_m_ decrease, 48%, in propofol glucuronidation by UGT1A9 that was expressed in HEK293 cells [6], whereas the use of 2% BSA instead of 0.1% had very little effect on darexaban glucuronidation by UGT1A9 that was expressed in Sf9 insect cells [7]. It is also worth pointing out that, for yet unclear reasons, previous reports on the BSA effects in UGT1A9 disagree on whether or not BSA addition increases reactions *V*_max_, regardless if 0.1, 1, or 2% BSA was used [6]–[10]. Taken together with the binding assays outcomes, our results indicate that the potential benefits of using 1% BSA are offset by much higher nonspecific binding of substrates, especially in the case of entacapone and estradiols (*f*_u_ <0.1), binding that would have prevented us from performing enzyme extensive kinetic assays with these substrates.

We then tested the BSA effects on 4-MU glucuronidation by UGT2A1, using different amounts of total UGT-enriched membrane protein, 0.02, 0.05, 0.1, 0.2, 0.5, and 1.0 mg/mL. The results revealed that 0.1% BSA similarly (and strongly) enhances the UGT2A1 activity in the presence of up to 0.5 mg/mL of total protein, while its effect in the presence of 1.0 mg/mL was lower. Taking this into account, in subsequent enzyme kinetic analyses we have avoided using any recombinant UGT at higher than 0.2 mg/mL of total sample protein.

### BSA Effects on the Enzyme Kinetics of Human UGTs

**Figure 2:**
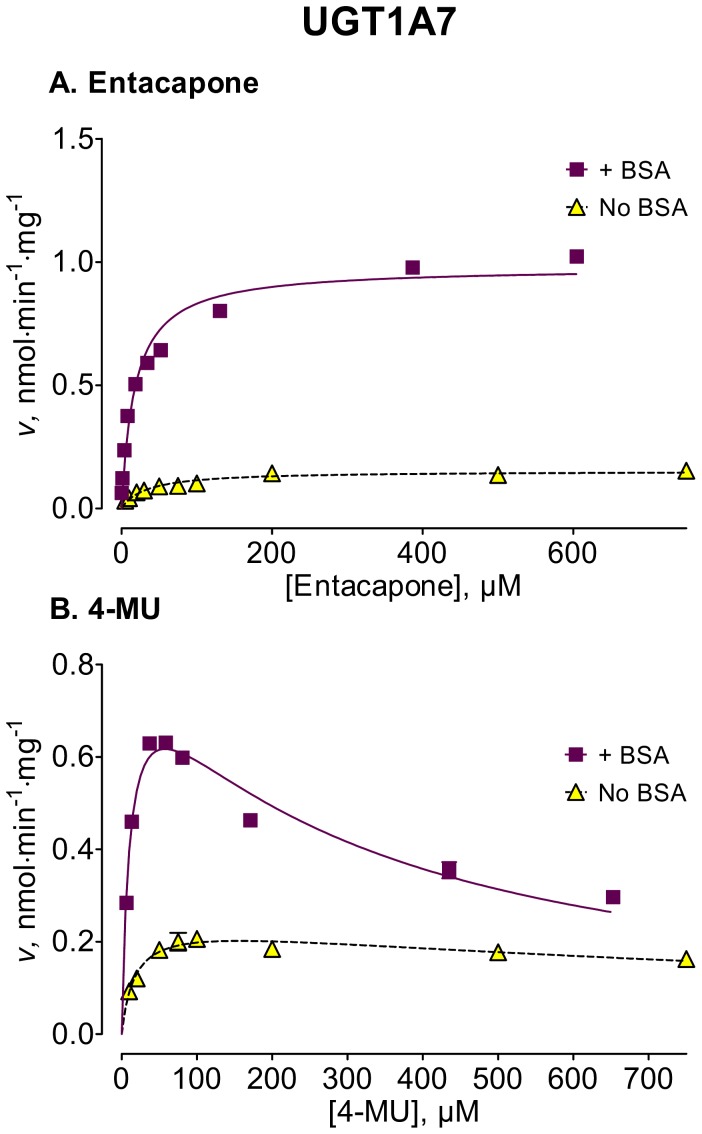
Enzyme kinetics of UGT1A7-catalyzed glucuronidation of entacapone (A) and 4-MU (B) in the absence and presence of BSA. The glucuronidation rates are presented as the average value ± S.E., and are expression level-normalized values (see *Materials and Methods*). The concentrations of substrates were corrected for binding to 0.1% BSA. The determined enzyme kinetic parameters are presented in Table 2.

**Figure 3:**
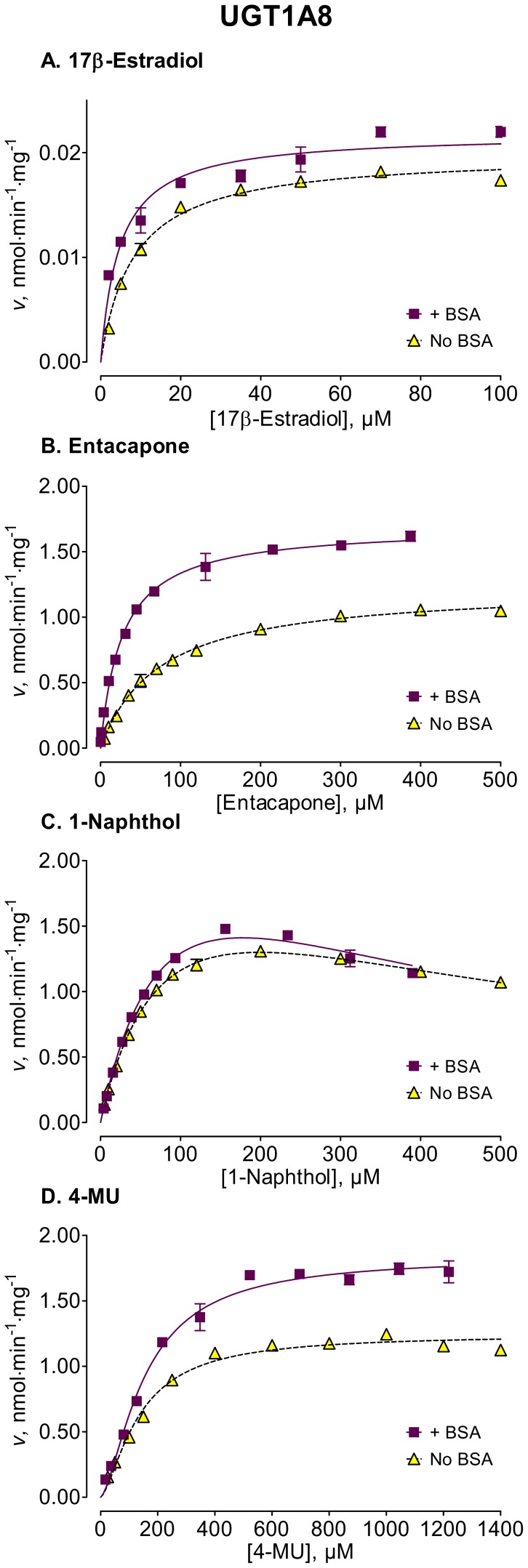
Enzyme kinetics of UGT1A8-catalyzed glucuronidation of 17β-estradiol (A), entacapone (B), 1-naphthol (C), and 4-MU (D) in the absence and presence of BSA. The reactions with 17β-estradiol were analyzed for the formation of 17β-estradiol-3-β-D-glucuronide. The glucuronidation rates are presented as the average value ± S.E., and are expression level-normalized values. The concentrations of substrates were corrected for binding to 0.1% BSA. The determined enzyme kinetic parameters are presented in Table 2. See *Materials and Methods* for all further details.

**Figure 4:**
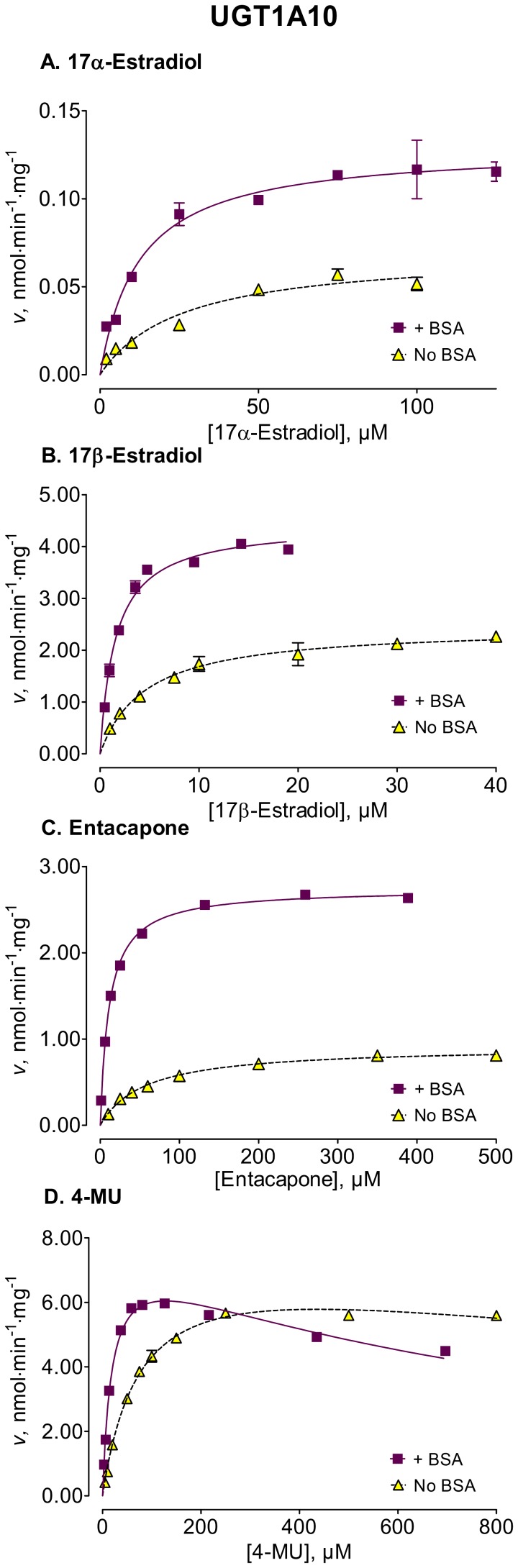
Enzyme kinetics of UGT1A10-catalyzed glucuronidation of 17α-estradiol (A), 17β-estradiol (B), entacapone (C), and 4-MU (D) in the absence and presence of BSA. The reactions with 17α-estradiol and 17β-estradiol were analyzed for the formation of 17α-estradiol-3-β-D-glucuronide and 17β-estradiol-3-β-D-glucuronide, respectively. The glucuronidation rates are presented as the average value ± S.E., and are expression level-normalized values. The concentrations of substrates were corrected for binding to 0.1% BSA. The determined enzyme kinetic parameters are presented in Table 2. See *Materials and Methods* for all further details.

**Figure 5:**
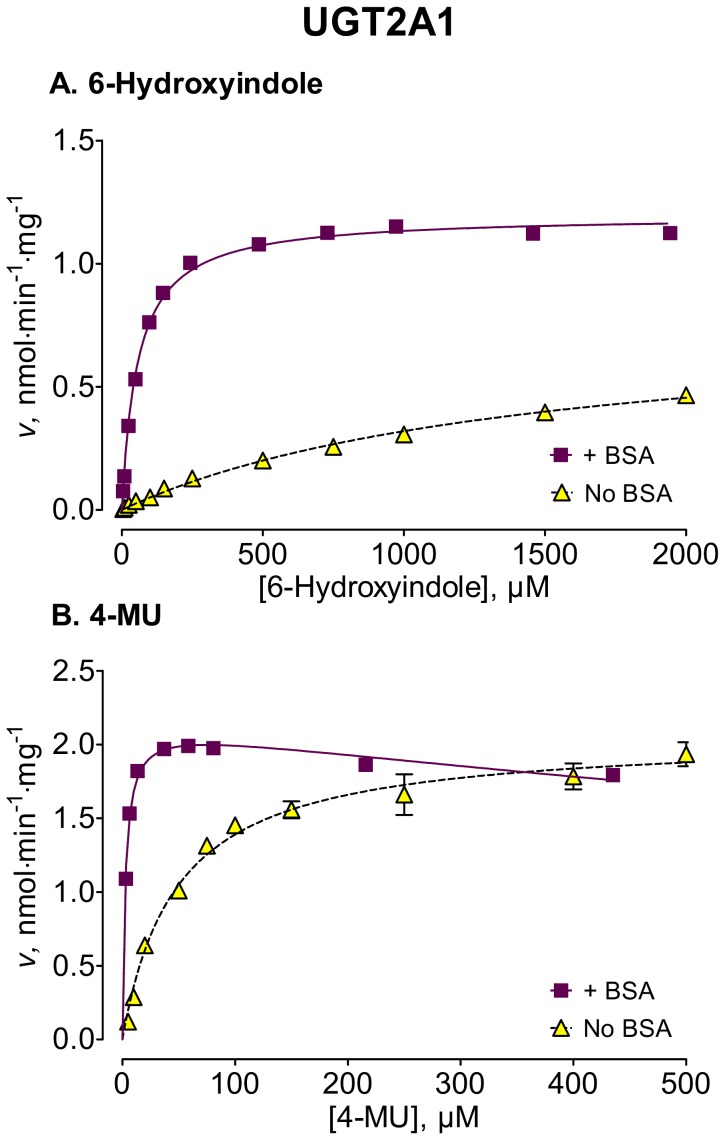
Enzyme kinetics of UGT2A1-catalyzed glucuronidation of 6-hydroxyindole (A) and 4-MU (B), in the absence and presence of BSA. The glucuronidation rates are presented as the average value ± S.E., and are expression level-normalized values. The concentrations of substrates were corrected for binding to 0.1% BSA. The determined enzyme kinetic parameters are presented in Table 3. See *Materials and Methods* for all further details.

**Figure 6:**
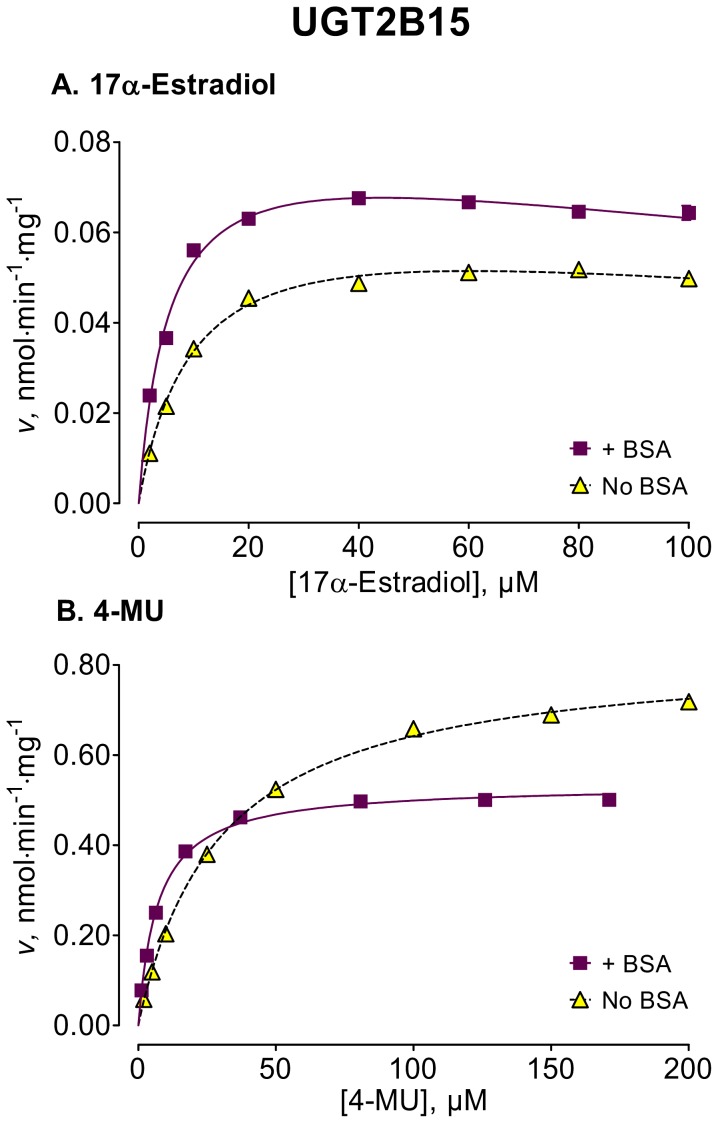
Enzyme kinetics of UGT2B15-catalyzed glucuronidation of 17α-estradiol (A) and 4-MU (B), in the absence and presence of BSA. The reaction with 17α-estradiol was analyzed for the formation of 17α-estradiol-3-β-D-glucuronide. The glucuronidation rates are presented as the average value ± S.E. These values are not expression normalized because we used commercial UGT2B15 without appropriate His-tag. The concentrations of substrates were corrected for binding to 0.1% BSA. The determined enzyme kinetic parameters are presented in Table 3. See *Materials and Methods* for all further details.

**Figure 7:**
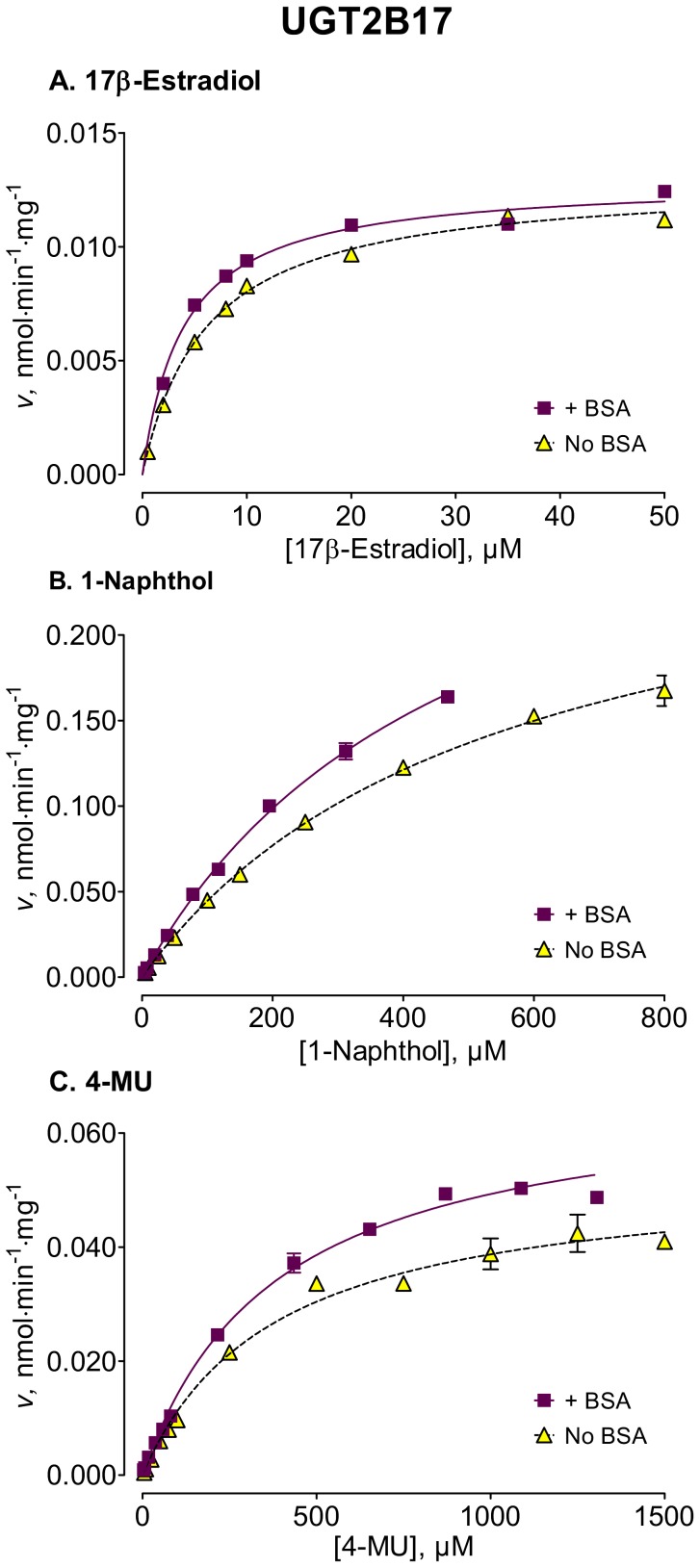
Enzyme kinetics of UGT2B17-catalyzed glucuronidation of 17β-estradiol (A), 1-naphthol (B), and 4-MU (C), in the absence and presence of BSA. The reaction with 17β-estradiol was analyzed for the formation of 17β-estradiol-17-β-D-glucuronide. The glucuronidation rates are presented as the average value ± S.E., and are expression level-normalized values. The concentrations of substrates were corrected for binding to 0.1% BSA. The determined enzyme kinetic parameters are presented in Table 3. See *Materials and Methods* for all further details.

**Figure 8:**
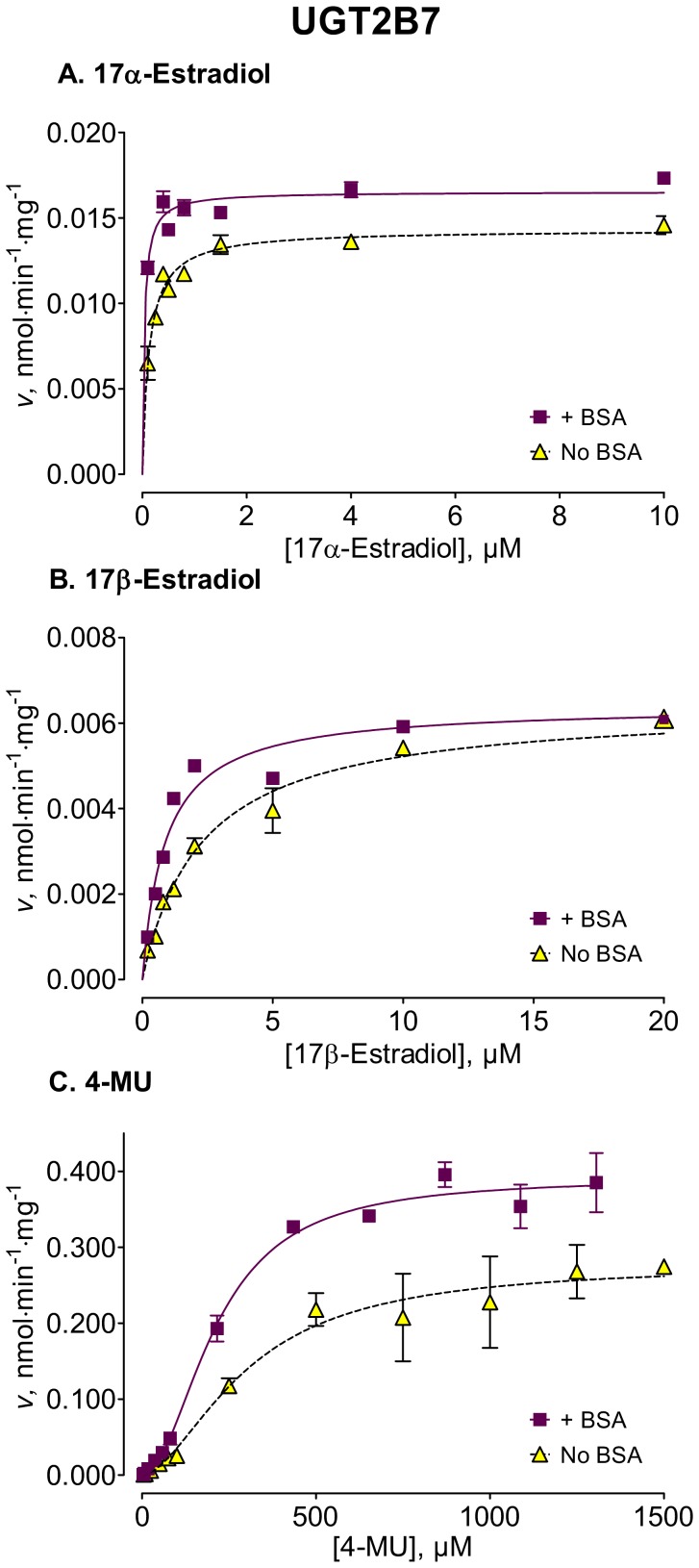
Enzyme kinetics of UGT2B7-catalyzed glucuronidation of 17α-estradiol (A), 17β-estradiol (B), and 4-MU (C), in the absence and presence of BSA. The reactions with 17α-estradiol and 17β-estradiol were analyzed for the formation of 17α-estradiol-17-β-D-glucuronide and 17β-estradiol-17-β-D-glucuronide, respectively. The glucuronidation rates are presented as the average value ± S.E., and are expression level-normalized values. The concentrations of substrates were corrected for binding to 0.1% BSA. The determined enzyme kinetic parameters are presented in Table 3. See *Materials and Methods* for all further details.

We used several new substrates to investigate the BSA effects on six UGT enzymes that have not been studied before, UGTs 1A7, 1A8, 1A10, 2A1, and 2B15, and 2B17, as well as four UGTs, 1A1, 1A6, 2B4, and 2B7, that were already examined [4]–[7], [10], [12]. The enzyme kinetic curves for the newly tested enzymes are presented in Figs. 2, 3, 4, 5, 6, 7, whereas Fig. 8 presents our assays for UGT2B7, the most studied UGT enzyme as far as the BSA effects are concern [4], [5], [10]. For the reason of clarity and limited space, the enzyme kinetic curves for the previously tested enzymes, UGT1A1, UGT1A6 and UGT2B4, are presented in supplementary material, even if these assay conditions, BSA concentration and substrates selection, differ between the new assays to the previous ones (Fig. S1, S2, S3). The calculated enzyme kinetic parameters for the UGTs of subfamily 1A are presented in Table 2, whereas the corresponding results for the UGTs of subfamilies 2A and 2B are given in Table 3.

**Table 2:** Enzyme kinetic parameters of UGTs 1A1, 1A6, 1A7, 1A8, and 1A10-catalyzed glucuronidation in the absence and presence of BSA.

				No BSA			0.1% BSA		
UGT enzyme/substrate				*K*_m_ or *S*_50_± S.E.(95% CI)	*V*_max_ ± S.E.(95% CI)	Model, *r* ^2^	*K*_m_ or *S*_50_± S.E.(95% CI)	*V*_max_ ± S.E.(95% CI)	Model, *r* ^2^
				*µM*	*nmol•min^–1^•mg^–1^*	*µM (S.E.)*	*µM*	*nmol•min^–1^•mg^–1^*	*µM (S.E.)*
UGT1A1									
17α-Estradiol (3-glucuronide)				4.57±0.48(3.59–5.56)	0.019±0.001(0.018–0.020)	MM, 0.97	4.79±0.28(4.21–5.37)	0.020±0.0003(0.019–0.020)	MM, 0.99
17β-Estradiol (3-glucuronide)				7.84±1.62(4.48–11.21)	0.19±0.01(0.17–0.21)	MM, 0.89	9.06±1.14(6.70–11.42)	0.24±0.01***(0.23–0.26)	MM, 0.96
1-Naphthol				101±11(78–123)	0.11±0.01(0.10–0.13)	HE, 0.96;*h* = 1.88 (0.33)	81.6±5.1(71.0–92.1)	0.17±0.01**(0.16–0.18)	HE, 0.98;*h* = 2.33 (0.31)
4-MU				72.6±6.9(58.2–87.0)	0.11±0.004(0.11–0.12)	MM, 0.98	64.2±8.4^†^(46.7–81.7)	0.25±0.02^†^(0.21–0.29)	SI, 0.99;*K*_i_ = 537 (115)
UGT1A6									
6-Hydroxyindole				390±78(227–553)	7.0±0.8(5.3–8.7)	SI, 0.98;*K*_i_ = 2800 (576)	300±40(217–382)	8.0±0.6(6.8–9.1)	SI, 0.98;*K*_i_ = 2989 (604)
1-Naphthol				8.07±0.48(7.08–9.06)	6.7±0.1(6.47–6.94)	MM, 0.99	5.25±0.23***(4.78–5.72)	5.9±0.1***(5.8–6.0)	MM, 0.99
4-MU				76.4±15.1(45.3–107.5)	5.2±0.3(4.5–6.0)	MM, 0.94	57.5±2.0(53.4–61.5)	9.6±0.1***(9.4–9.9)	MM, 0.99
UGT1A7									
Entacapone			33.2±4.0(24.9–41.6)		0.15±0.01(0.14–0.16)	MM, 0.93	17.9±1.9(14.0–21.7)	0.98±0.03***(0.93–1.03)	MM, 0.96
4-MU			17.6±3.1(11.0–24.2)		0.25±0.01(0.22–0.28)	SI, 0.86;*K*_i_ = 1398(339)	11.5±1.4(8.4–14.5)	0.86±0.04***(0.78–0.94)	SI, 0.95;*K*_i_ = 289 (34)
UGT1A8									
17β-Estradiol (3-glucuronide)		8.16±0.55(7.03–9.30)			0.020±0.0003(0.019–0.021)	MM, 0.99	4.70±0.62**(3.42–5.99)	0.022* ±0.001(0.021–0.023)	MM, 0.95
Entacapone		73.1±3.7(65.4–80.7)			1.2±0.02(1.2–1.3)	MM, 0.99	26.9±1.7***(23.8–30.1)	1.7±0.02***(1.6–1.8)	MM, 0.99
1-Naphthol		91.6±6.6(78.0–105.3)			2.5±0.1(2.3–2.7)	SI, 0.99;*K*_i_ = 435 (40)	105±16(73–137)	3.1±0.3**(2.4–3.7)	SI, 0.99;*K*_i_ = 300 (55)
4-MU		134±9(115–154)			1.2±0.03(1.2–1.3)	HE, 0.98;*h* = 1.49 (0.14)	154±11(132–176)	1.8±0.1***(1.7–2.0)	HE, 0.99;*h* = 1.52 (0.14)
UGT1A10									
17α-Estradiol (3-glucuronide)	25.7±6.3(12.8–39.1)				0.070±0.006(0.058–0.082)	MM, 0.92	12.3±1.8*(8.5–16.1)	0.13±0.01**(0.12–0.14)	MM, 0.95
17β-Estradiol (3-glucuronide)	4.58±0.61(3.30–5.85)				2.5±0.1(2.3–2.7)	MM, 0.93	1.56±0.11***(1.33–1.80)	4.4±0.1***(4.27–4.60)	MM, 0.98
Entacapone	56.7±2.3(51.9–61.5)				0.91±0.01(0.89–0.94)	MM, 0.99	11.2±0.3***(10.6–11.9)	2.7±0.02***(2.7–2.8)	MM, 0.99
4-MU	80.7±6.6(67.0–94.3)				8.0±0.3(7.3–8.7)	SI, 0.99;*K*_i_ = 2289 (425)	19.2±1.3***(16.6–21.8)	7.8±0.2(7.48–8.22)	SI, 0.99;*K*_i_ = 855 (71)

The glucuronidation rates are presented as expression level-normalized values ± S.E. The 95% CI are presented in the parenthesis. The p-values were calculated using the extra sum-of-squares F-test (see *Materials and Methods* for all details).

MM, Michaelis-Menten; SI, substrate inhibition; HE, Hill equation;

* P<0.05;

** P<0.01;

*** P<0.001.

**Table 3:** Enzyme kinetic parameters of UGTs 2A1, 2B4, 2B7, 2B15, and 2B17-catalyzed glucuronidation in the absence and presence of BSA.

	No BSA			0.1% BSA		
UGT enzyme/substrate	*K*_m_ or *S*_50_± S.E. (95% CI)	*V*_max_ ± S.E. (95% CI)	Model, *r* ^2^	*K*_m_ or *S*_50_± S.E. (95% CI)	*V*_max_ ± S.E. (95% CI)	Model, *r* ^2^
	*µM*	*nmol•min^–1^•mg^–1^*	*µM (S.E.)*	*µM*	*nmol•min^–1^•mg^–1^*	*µM (S.E.)*
UGT2A1						
6-Hydroxyindole	1474±121(1223–1724)	0.79±0.04(0.72–0.87)	MM, 0.99	56.6±2.3***(51.8–61.4)	1.2±0.01***(1.17–1.22)	MM, 0.99
4-MU	48.1±4.8(38.2–57.9)	2.1±0.1(1.9–2.1)	MM, 0.97	2.73±0.20^†^(2.41–3.05)	2.1±0.02^†^(2.1–2.2)	SI, 0.97;*K*_i_ = 2045 (255)
UGT2B4						
17α-Estradiol (17-glucuronide)	4.51±0.68(3.09–5.93)	0.053±0.002(0.048–0.058)	MM, 0.95	1.60±0.23***(1.12–2.08)	0.047±0.002(0.044–0.050)	MM, 0.91
1-Naphthol	210±12(186–235)	0.025±0.001(0.023–0.027)	HE, 0.96;*h* = 2.62 (0.36)	99.8±7.5***(84.3–115.4)	0.0080±0.0032***(0.0074–0.0087)	HE, 0.89;*h* = 4.13 (1.00)
4-MU	298±43(210–387)	0.043±0.002(0.039–0.047)	MM, 0.95	192±15*(161–222)	0.035±0.001***(0.033–0.037)	MM, 0.98
UGT2B7						
17α-Estradiol (17-glucuronide)	0.128±0.015(0.098–0.158)	0.014±0.0003(0.014–0.015)	MM, 0.96	0.0379±0.0065***(0.0242–0.0515)	0.016±0.0003***(0.016–0.017)	MM, 0.97
17β-Estradiol (17-glucuronide)	2.56±0.29(1.96–3.16)	0.0067±0.0003(0.0062–0.0072)	MM, 0.96	0.832±0.096***(0.633–1.031)	0.0063±0.0002(0.0059–0.0067)	MM, 0.94
4-MU	301±65(165–436)	0.28±0.03(0.22–0.34)	HE, 0.93; *h* = 1.75(0.46)	212±17(177–247)	0.39±0.01*(0.36–0.42)	HE, 0.99;*h* = 2.01 (0.24)
UGT2B15						
17α-Estradiol (3-glucuronide)	10.2±1.0(8.0–12.3)	0.069±0.003(0.062–0.076)	SI, 0.99;*K*_i = _360 (92)	5.58±0.56***(4.24–6.74)	0.085±0.003**(0.077–0.092)	SI, 0.98;*K*_i_ = 348 (80)
4-MU	29.6±0.9(27.6–31.5)	0.83±0.01(0.82–0.85)	MM, 0.99	7.06±0.30***(6.54–7.54)	0.53±0.01***(0.53–0.54)	MM, 0.99
UGT2B17						
17β-Estradiol (17-glucuronide)	6.01±0.26(5.47–6.54)	0.013±0.0002(0.0125–0.0132)	MM, 0.99	4.01±0.24***(3.51–4.51)	0.013±0.0002(0.0126–0.0134)	MM, 0.98
1-Naphthol	540±26(485–595)	0.28±0.01(0.26–0.31)	MM, 0.99	484±27(427–541)	0.34±0.02**(0.30–0.37)	MM, 0.99
4-MU	377±42(291–463)	0.050±0.002(0.049–0.058)	MM, 0.99	383±29(322–444)	0.068±0.002 ***(0.064–0.072)	MM, 0.99

The glucuronidation rates are presented as expression level-normalized values ± S.E., except for the commercial UGT2B15. The 95% CI are presented in the parenthesis. The p-values were calculated using the extra sum-of-squares F-test (see *Materials and Methods* for all details).

MM, Michaelis-Menten; SI, substrate inhibition; HE, Hill equation;

* P<0.05;

** P<0.01;

*** P<0.001.

The principal aim of the current enzyme kinetics assays was to examine the influence of BSA addition on substrate affinity (*K*_m_ or *S*_50_) and reaction limiting velocity (*V*_max_). Due to the large number of experiments, significant differences in the assays with and without BSA, as well as the common appearance of substrate inhibition or sigmoidal kinetics, we could not examine each individual reaction in full detail. As a result, the determined enzyme kinetic parameters may be regarded as close estimates, especially in the case of very high affinity substrates (17α-estradiol glucuronidation by UGT2B7) or reactions that follow sigmoidal saturation profile. The detailed mechanistic understanding of reactions that exhibit sigmoidal kinetics may require the use of multi-site kinetic models [27]–[30], a topic that is outside of the focus of the current study.

In order to ascertain the statistical significance of the found differences between enzyme kinetic parameters determined in the absence and presence of BSA, we performed an extra sum-of-squares F-test for each pair of parameters. The result of this statistical analysis, as well as the calculated p-values for the respective *K*_m_ and *V*_max_ pairs, are presented in Tables 2 and 3. In addition, for a comparison of BSA affects across different UGTs, we have presented the relative changes of kinetic parameters in different UGTs, in the glucuronidation of different substrates, in a supplementary figure (Fig. S4). For this broad comparison we calculated the average relative *K*_m_, *V*_max_, and CL_int_ or CL_max_ in the presence of BSA, as well as the corresponding propagated S.E. of the average that includes the errors of enzyme kinetic parameters from the reactions in both the presence and absence of BSA (see *Materials and Methods* for further details). In some assays, however, especially in the cases of 4-MU and 1-naphthol glucuronidation, the enzyme kinetics was best described by sigmoidal model (eq. 3) and, therefore, the *S*_50_ value, not the *K*_m_, was taken as a measure for substrate affinity. Nevertheless, for simplicity and in order to present the overall picture of the relative changes in substrate affinity due to the presence of 0.1% BSA, the relative *K*_m_ and *S*_50_ changes in Fig. S4 were combined.

### Enzyme Kinetic Parameters in the Absence of BSA

The results revealed that the enzyme kinetic parameters, for the tested UGTs, in the absence of BSA (Tables 2 and 3) were mainly similar to those in the previous reports on 17α- and 17β-estradiol [24], 4-MU and 1-naphthol [27], [31], and entacapone glucuronidation [32]. In most cases, but not all, the addition of 0.1% BSA influenced the enzyme kinetics of the tested enzymes. The exact effects, however, were dependent on both the tested enzyme and the used substrate.

### BSA Effects on the *K*_m_ (or *S*_50_) Values of UGT-catalyzed Reactions

Among the newly tested enzymes, the inclusion of 0.1% BSA led to significant decreases of *K*_m_ values in UGTs 1A7, 1A8, 1A10, 2A1, and 2B15 (kinetic curves in Figs. 2, 3, 4, 5, 6, parameters in Tables 2 and 3). The effect was most pronounced in UGT2A1 (up to 20-fold) followed by UGT1A10 (Table 2). The *K*_m_ values of 2B17, the last previously untested enzyme, were much less affected, exhibiting a significant but mild decrease only with one substrate, 17β-estradiol (Fig. 7, Table 3).

In many cases the BSA effects on the *K*_m_ values appeared relatively independent of the used substrate, but deviation from this rule was observed in UGT2B15, UGT2B17, and especially UGT1A8. For UGT1A8 the addition of BSA sharply decreased *K*_m_ value when assayed for the glucuronidation of the relatively large substrates, entacapone and 17β-estradiol (Fig. 1), whereas no *K*_m_ decrease was found for UGT1A8 when the glucuronidation of the smaller substrates, 4-MU and 1-naphthol, was examined (Fig. 3 and Table 2).

In the group of previously tested enzymes, the inclusion of 0.1% BSA led to significant *K*_m_ value decreases in UGTs 2B4 and 2B7 (Table 3), whereas the *K*_m_ values of UGTs 1A1 and 1A6 were relatively unaffected (Table 2). These results are in good agreement with previous reports on the BSA effects in UGT2B4 [12], UGT2B7 [4], [5], as well as UGTs 1A1 and 1A6 [6], all of which employed 2% BSA.

### BSA Effects on the *V*_max_ Values

The BSA effects on the *V*_max_ values of the different UGTs appeared unrelated to the corresponding effects on the *K*_m_ values (kinetic curves in Figs. 2, 3, 4, 5, 6, 7, Tables 2 and 3, summary in Fig. S4). Significant, sometime large, *V*_max_ increases were observed and, interestingly, they were substrate-dependent in most cases. The highest *V*_max_ increase was found in UGT1A7 (Fig. 2, Table 1), up to 7-fold. Significant but strongly substrate-dependent *V*_max_ increases were found in UGT1A1 and UGT1A10 (Table 2), whereas in UGTs 1A8, 2A1, 2B4, 2B7, 2B15 and 2B17 no large *V*_max_ increases were observed with any of the 2–3 tested substrates (Table 2 and 3).

### BSA Effects on the CL_int_ and CL_max_ Values

The BSA-induced changes in the CL_int_ values of the different reactions were mostly large (Fig. S4, note the log_10_ of the y-axis scale). The CL_int_, in addition to simply being the *V*_max_/*K*_m_ ratio, may also be regarded as a fundamental parameter of the Michaelis-Menten equation, with the physical meaning of a second-order rate constant for the overall glucuronidation reaction at low substrate concentrations. Examining the BSA effect on this constant is interesting since it is affected by changes in both the *V*_max_ and the *K*_m_ values. It should be noted, however, that in the case of substrates that follow sigmoidal kinetics (eq. 3) we calculated the CL_max_, the maximal clearance that results from autoactivation (eq. 5) [22].

From the perspective of CL_int_ increase, UGT2A1 and UGT1A7 are in a group of enzymes that are highly affected by BSA, even if they differ largely in the kinetic constants that are most affected by BSA addition (*K*_m_ in UGT2A1, but mainly *V*_max_ in UGT1A7).

A plot of the BSA effect on the *K*_m_ values against the corresponding effects on the *V*_max_ values (Fig. S5) revealed no meaningful correlation between the BSA effects on the two kinetic parameters.

### Possible Effects of Assay Conditions on the Observed BSA Effects

In carrying out the large number of glucuronidation reactions that were done in this study, it is possible that some samples were handled somewhat differently from others. Hence, the large and substrate-dependent BSA effects in some cases, prompted us to examine whether or not there is a correlation between the BSA effects and the assay conditions. The results of these experiments are presented in the supplementary materials (Fig. S6) and they show no correlation between the magnitude of the BSA effects and assay conditions, such as the incubation time or total concentration of UGT protein during the reaction.

## Discussion

BSA addition enhances the in vitro activities of several UGTs and improves in vitro–in vivo extrapolation [4]–[10], [12], [33], [34]. Not enough, however, is known and understood about the extent and mechanism of the BSA effects. The principle goal of this work was to investigate these effects in six previously untested UGT enzymes, UGTs 1A7, 1A8, 1A10, 2A1, 2B15, and 2B17, as well as to reexamine part of the previously studied BSA effects on UGTs 1A1, 1A6, 2B4, and 2B7 with a more diverse set of substrates in order to find out whether or not the BSA effects are dependent on the used substrate.

Six different UGT substrates, 17α-estradiol, 17β-estradiol, entacapone, 6-hydroxyindole, 1-naphthol, and 4-MU, were used for enzyme kinetic assays, in both the absence and presence of BSA. The results were calculated following the determination of their *f*_u_ in the presence of 0.1% BSA and the used enzymes source. The selected UGT substrates have different physicochemical properties and a wide distribution of enzyme kinetic parameters, providing together a good representation of the chemical space that is typically encountered by UGT enzymes. Most of the selected substrates allow for sensitive detection in HPLC analyses (see Table 1 for the limits of detection and quantification), enabling assays with relatively low concentrations of the UGT-enriched membrane, up to 0.2 mg/mL of total protein. Under these conditions, the inclusion of 0.1% BSA was sufficient to achieve good stimulatory effects in most cases [7]–[9].

Although assays with 1% BSA, in comparison with assays including 0.1% BSA, resulted in somewhat better enhancement of enzyme activity (see *Results* section), the use of 1% BSA has the disadvantage of high nonspecific binding of substrates. The use of 0.1% BSA, rather than 1 or 2%, significantly lowered the nonspecific binding and enabled us to carry out more accurately assays with physiologically relevant compounds, such as estradiol and the therapeutic drug entacapone. Although clearly a compromise, this strategy offers an advantage over assays that combine high concentrations of albumin (1 or 2%) with hydrophobic UGT substrates or inhibitors. For example, Walsky et al. (2012) performed enzyme kinetic assays with 17β-estradiol in the presence of 2% BSA, conditions under which the *f*_u_ of 17β-estradiol is very low, barely 0.04. A similar problem was encountered in a substrate depletion study with a number of hydrophobic drugs [34], as well as in inhibition assays with spironolactone [35] and niflumic acid [36]. In the latter case BSA was omitted from the assays due to excessive nonspecific binding of the inhibitors. Taken together, we think that the use of 0.1% BSA is a reasonable compromise between good activation of UGT enzymes and relatively low nonspecific binding of substrates to BSA. An alternative solution for high nonspecific binding of substrates and inhibitors may be the previously suggested replacement of BSA with intestinal fatty acid binding protein [25]. The advantage of this expensive alternative over the use of 0.1% BSA, however, has still to be tested with more enzymes and substrates.

The addition of 0.1% BSA significantly enhanced the activities of most human UGTs. Nevertheless, it is worth noting that there was a significant difference in the routes that lead to this enhancement, as well as in its degree. In some of the enzymes, namely the stimulation was through a *K*_m_ decrease (UGTs 1A7, 1A8, 1A10, 2A1, and 2B15 of the UGTs that were not previously tested) that was accompanied by *V*_max_ increases in two of them, UGT1A7 and UGT1A10 (Figs. 2, 3, 4, 5, 6, 7, Tables 2 and 3).

It is also important to note that there is a clear variability in the BSA effects on the human UGTs. For example, while UGTs 2B7 and 2B17 interact with similar type of substrates, estradiols, the BSA effects on UGT2B7 are much larger than on UGT2B17. In addition, with 4-MU as the substrate, we have found a significant *V*_max_ increase in both UGT1A1 and 1A6, whereas in the case of 1-naphthol glucuronidation the inclusion of BSA in the reaction mixture increased the *V*_max_ value of UGT1A1, but not UGT1A6 (Figs. S1 and S2, Table 2).

Although our understanding of the *K*_m_ and *V*_max_ changes is important for mechanistic studies of BSA effects, the overall magnitude of activity enhancement, especially at low concentrations of substrate, may be more relevant for studies focused on in vitro–in vivo extrapolation. Such an overall activity enhancement in the presence of BSA may be better expressed through relative changes in CL_int_ or, in the case of sigmoidal kinetics, CL_max_ (Fig. S4, note the log_10_ scale of the y-axis). These changes can simply be interpreted as the combined effects of *K*_m_ and *V*_max_ modulation, or, from a slightly different point of view, as the overall enhancement of enzyme activity at low concentrations of substrate. The results show that inclusion of 0.1% BSA leads to large CL_int_ increases in UGTs 1A7, 1A10, and 2A1. The CL_int_ increase, up to 400-fold, is especially striking in the case of UGT2A1, an enzyme with broad substrate selectivity, but limited tissue expression pattern [37].

The BSA effects on the *K*_m_ values of some, but not all, UGTs raises questions about the nature of the BSA effects. If the apparently higher affinity for the substrates in the presence of the BSA is due to BSA-mediated removal of lipid inhibitors, such as arachidonic or linoleic acid [5], then the lack of such effects in UGTs 1A1, 1A6, and 2B17 would mean that these enzymes are not inhibited by such inhibitors, at least not in the way that compromises the binding of aglycone substrate. It is also worth noting that the inclusion of BSA decreases the *K*_m_ values of the affected enzymes, even if the substrate affinity is already very high, as in the case of 17α-estradiol and UGT2B7 (Fig. 8, Table 3). We recently observed similar phenomena with 1-naphthol and UGT1A9 [9]. Although surprising as first glance, these results are generally consistent with the theory of competitive inhibition, in particular with the fact that the *K*_mI_/*K*_m_ ratio, the ratio of apparent substrate affinities in the presence (*K*_mI_) and absence (*K*_m_) of the inhibitor, is solely dependent on the [*I*]/*K*_i_ ratio, rather than on the absolute magnitude of the substrate affinity. In line with this, the tentative UGT inhibitors that are removed by BSA, such as fatty acids, may exhibit very high affinity toward the enzymes [5], [38], [39].

The results with UGT1A8 are of particular interest since in this enzyme BSA addition led to *K*_m_ decrease when the larger substrates, 17β-estradiol and entacapone, were used, but no changes when 1-naphthol and 4-MU, much smaller molecules (Fig. 1), were used as the glucuronidation substrates (Fig. 3, Table 2). This might indicate that the two substrate groups have unique binding modes, each differently affected by BSA addition. Additional studies will be needed to fully resolve this phenomenon and find out if it could be documented in other human UGTs.

In contrast to the BSA effects on the *K*_m_ values that, with the exception of UGT1A8, were not highly dependent on the used substrate, the BSA effects on *V*_max_ values were significantly influenced by the used substrate (Figs. 2, 3, 4, 5, 6, 7, 8, Tables 2 and 3). A *V*_max_ increase with all tested substrates was only found in the present study with UGT1A7 and in other studies with UGT1A9 [8], [9] (Fig. 2, Table 1). The considerable BSA-induced *V*_max_ increases in UGTs 1A1, 1A6, and 1A10 were clearly substrate dependent (Table 2). These results may indicate that BSA-induced *V*_max_ increases are mainly limited to the UGT1A subfamily.

The *V*_max_ value increase in the presence of BSA may be explained by albumin-mediated removal of noncompetitive of mixed-type inhibitors with respect to the aglycone substrate [9]. Taking into account the compulsory-order of substrate binding in UGT-catalyzed reaction [9], [31], such inhibitors would also bind to the ternary enzyme•UDPGA•aglycone complex. As structurally different aglycone substrates may lead to slightly different ternary complexes, these tentative UGT inhibitors may have different affinities for them, explaining the differential effects of BSA inclusion on *V*_max_ values. Moreover, no correlation between relative *K*_m_ and *V*_max_ changes was found in this study (Fig. S5), suggesting that the possible removal of different inhibitors is responsible for these two independent effects. Considering the chemical structure of these putative “*V*_max_-affecting” inhibitors, no data is currently available, but it could well be one or more of the fatty acids that bind to BSA.

Our previous study on zidovudine and entacapone glucuronidation in the presence of BSA clearly demonstrated that there are no major differences in the BSA effects between UGTs in HLM and recombinant enzymes that were expressed in Sf9 insect cells [8]. Hence, the presence in Sf9 cells of less linoleic and arachidonic acid, but more oleic and palmitoleic acid than HLM [5], [40] does not change the BSA effects significantly. Therefore, the current findings on the BSA effects in the recombinant UGTs (expressed in Sf9 insect cells) are expected to represent, both qualitatively and quantitatively, the corresponding BSA effects in HLM. However, depending on the total amount of an enzyme source in the assay, the optimal concentration of added BSA may require further adjustments, especially if more than 0.2 mg/mL total membrane protein is used, the highest concentration of enzyme source that we have used in this work.

The mostly comparable BSA effects that were so far found for UGTs in HLM and recombinant systems, either HEK293 or Sf9 cells, may be different from the corresponding BSA effects in cytochrome P450 (CYP) assays, however. The latter is suggested based on reports that the BSA effects in recombinant CYPs 1A2, 2C8, and 2C9 were less pronounced compared to assays with HLM [41]–[43]. Nevertheless, these recombinant CYPs could not be compared to the recombinant UGTs since they were expressed in bacterial cells, whereas the recombinant UGTs are expressed in eukaryotic cells. Unfortunately, there is currently no good way to express recombinant human UGTs in *E. coli* cells, the system used for recombinant CYP enzymes expression, and it is impossible to test in what way the lower abundance of inhibitory fatty acids in these bacterial cells [43] affects the UGTs and their activity stimulation by BSA.

In conclusion, we have described the BSA effects in six previously unexamined human UGTs 1A7, 1A8, 1A10, 2A1, 2B15 and 2B17, and expanded previous findings on the BSA effects in UGTs 1A1, 1A6, 2B4, and 2B7. The combined results highlight the complexity and variability of this effect, far different from the original results with UGT2B7 where the BSA effect is restricted to the reaction *K*_m_ value [5]. BSA addition often increases the reaction's *V*_max_, an effect that appears to be dependent on both the aglycone substrate and the individual UGT. In addition, there are cases where BSA addition does not lead to significant activity stimulation or changes in its kinetic parameters. Taking all the results into account, this work expands the current knowledge on the different BSA effects, the function and, perhaps, also the structure of the human UGTs, providing important results and analysis for future studies on in vitro UGT assays, as well as further studies on the exact nature and mechanism of the inhibitors that are removed by BSA.

## Supporting Information

Figure S1 **Enzyme kinetics of UGT1A1-catalyzed glucuronidation of 17α-estradiol (A), 17β-estradiol (B), 1-naphthol (C), and 4-MU (D), in the absence and presence of BSA.** The reactions with 17α-estradiol and 17β-estradiol were analyzed for the formation of 17α-estradiol-3-β-D-glucuronide and 17β-estradiol-3-β-D-glucuronide, respectively. The glucuronidation rates are presented as the average value ± S.E., and are expression level-normalized values. The concentrations of substrates were corrected for binding to 0.1% BSA. The determined enzyme kinetic parameters are presented in Table 2. See *Materials and Methods* for all further details.
(TIF)

Figure S2 **Enzyme kinetics of UGT1A6-catalyzed glucuronidation of 6-hydroxyindole (A), 1-naphthol (B), and 4-MU (C), in the absence and presence of BSA.** The glucuronidation rates are presented as the average value ± S.E., and are expression level-normalized values. The concentrations of substrates were corrected for binding to 0.1% BSA. The determined enzyme kinetic parameters are presented in Table 2. See *Materials and Methods* for all further details.
(TIF)

Figure S3 **Enzyme kinetics of UGT2B4-catalyzed glucuronidation of 17α-estradiol (A), 1-naphthol (B), and 4-MU (C), in the absence and presence of BSA.** The reaction with 17α-estradiol was analyzed for the formation of 17α-estradiol-17-β-D-glucuronide. The glucuronidation rates are presented as the average value ± S.E., and are expression level-normalized values. The concentrations of substrates were corrected for binding to 0.1% BSA. The determined enzyme kinetic parameters are presented in Table 3. See *Materials and Methods* for all further details.
(TIF)

Figure S4 **The combined effects of BSA of the** ***K*** **_m_ (A),** ***V*** **_max_ (B), and CL_int_ or CL_max_ (C) of the ten tested human UGTs.** The average values of the enzyme kinetic parameters, determined in the absence of BSA, where arbitrary assigned to 100%, and the average corresponding values of *K*_m_, *V*_max_, and CL_int_ or CL_max_ in the presence of BSA were compared to the values in the absence of BSA and plotted for all the ten tested UGT enzymes. The presented errors are propagated S.E. values that take into account the errors in the parameters determined in both the absence and presence of BSA (See *Materials and Methods* for further details). Due to large increases of CL_int_ or CL_max_ in the presence of BSA, the values of y-axis in panel C are presented on a log_10_ scale.
(TIF)

Figure S5 **The correlation between relative** ***K*** **_m_ and** ***V*** **_max_ values in the presence of 0.1% BSA.** The average values of enzyme kinetic parameters, determined in the absence of BSA, where arbitrary assigned to 100%, and the average relative values of *K*_m_ and *V*_max_ in the presence of 0.1% BSA were plotted against each other.
(TIF)

Figure S6 **The correlation between relative enzyme kinetic parameters in the presence of 0.1% BSA (** ***K*** **_m_,** ***V*** **_max_, and CL_int_ or CL_max_) and experimental conditions of enzyme kinetic assays, namely incubation time (panels A–C) and total protein concentration (panels D–E).** The average values of enzyme kinetic parameters, determined in the absence of BSA, were arbitrary assigned to 100%, and the average relative values of *K*_m_, *V*_max_, and CL_int_ or CL_max_ in the presence of 0.1% BSA were plotted against experimental conditions of the corresponding enzyme kinetic assay. For this analysis, we combined in a single graph panel the average relative values of enzyme kinetic parameters (in the presence of BSA) from all ten UGT enzymes and six substrates. Due to large increases of CL_int_ or CL_max_ in the presence of 0.1% BSA, the values of y-axis are presented on log_10_ scale. The incubation time varied from 10 to 60 min (panels A–C), and UGT protein concentration, referring to total protein concentration in the membrane, varied from 0.02 to 0.2 mg/mL. See *Materials and Methods* for all further details.
(TIF)
