# Supplementary figures and images for: Albumin Stimulates the Activity of the Human UDP-Glucuronosyltransferases 1A7, 1A8, 1A10, 2A1 and 2B15, but the Effects Are Enzyme and Substrate Dependent
Source: PLoS One. 2013 Jan 23;8(1):e54767. doi: 10.1371/journal.pone.0054767 (PMC3553014; doi:10.1371/journal.pone.0054767)

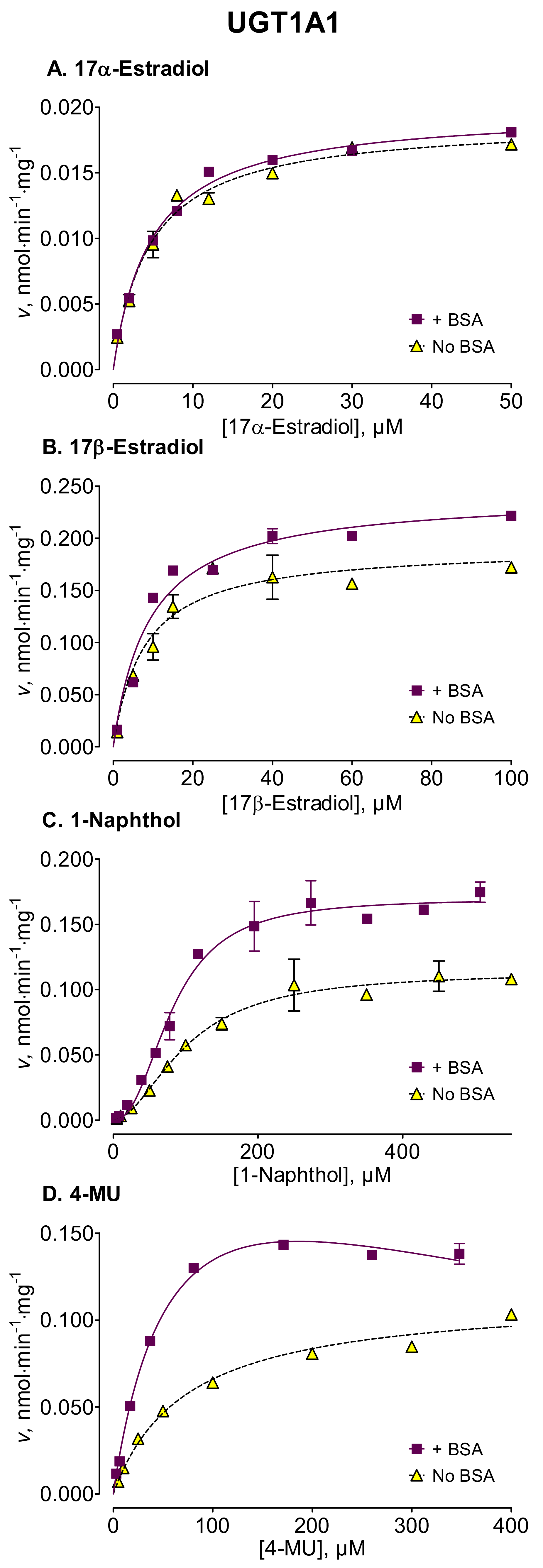

Supplement: Figure S1 — Enzyme kinetics of UGT1A1-catalyzed glucuronidation of 17α-estradiol (A), 17β-estradiol (B), 1-naphthol (C), and 4-MU (D), in the absence and presence of BSA. The reactions with 17α-estradiol and 17β-estradiol were analyzed for the formation of 17α-estradiol-3-β-D-glucuronide and 17β-estradiol-3-β-D-glucuronide, respectively. The glucuronidation rates are presented as the average value ± S.E., and are expression level-normalized values. The concentrations of substrates were corrected for binding to 0.1% BSA. The determined enzyme kinetic parameters are presented in Table 2. See Materials and Methods for all further details. (TIF) [file pone.0054767.s001.tif]

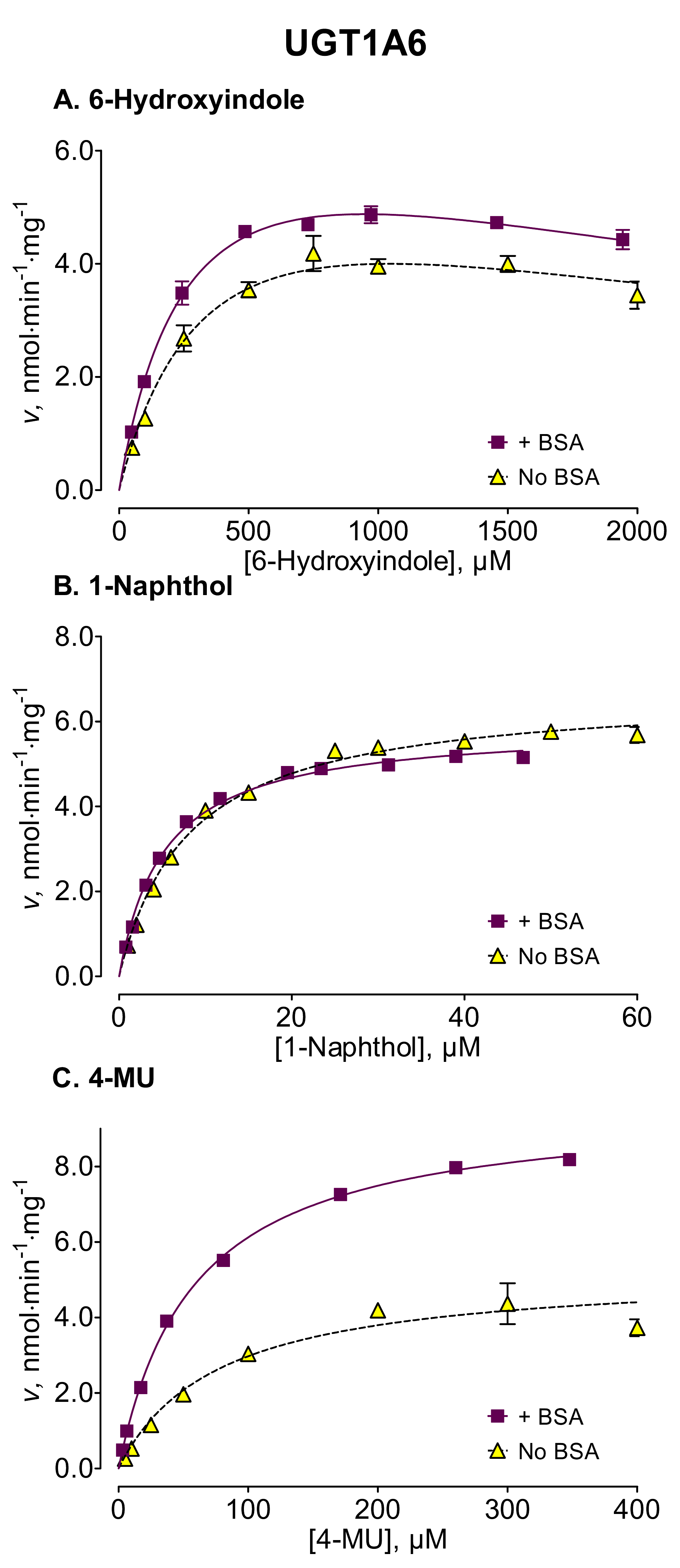

Supplement: Figure S2 — Enzyme kinetics of UGT1A6-catalyzed glucuronidation of 6-hydroxyindole (A), 1-naphthol (B), and 4-MU (C), in the absence and presence of BSA. The glucuronidation rates are presented as the average value ± S.E., and are expression level-normalized values. The concentrations of substrates were corrected for binding to 0.1% BSA. The determined enzyme kinetic parameters are presented in Table 2. See Materials and Methods for all further details. (TIF) [file pone.0054767.s002.tif]

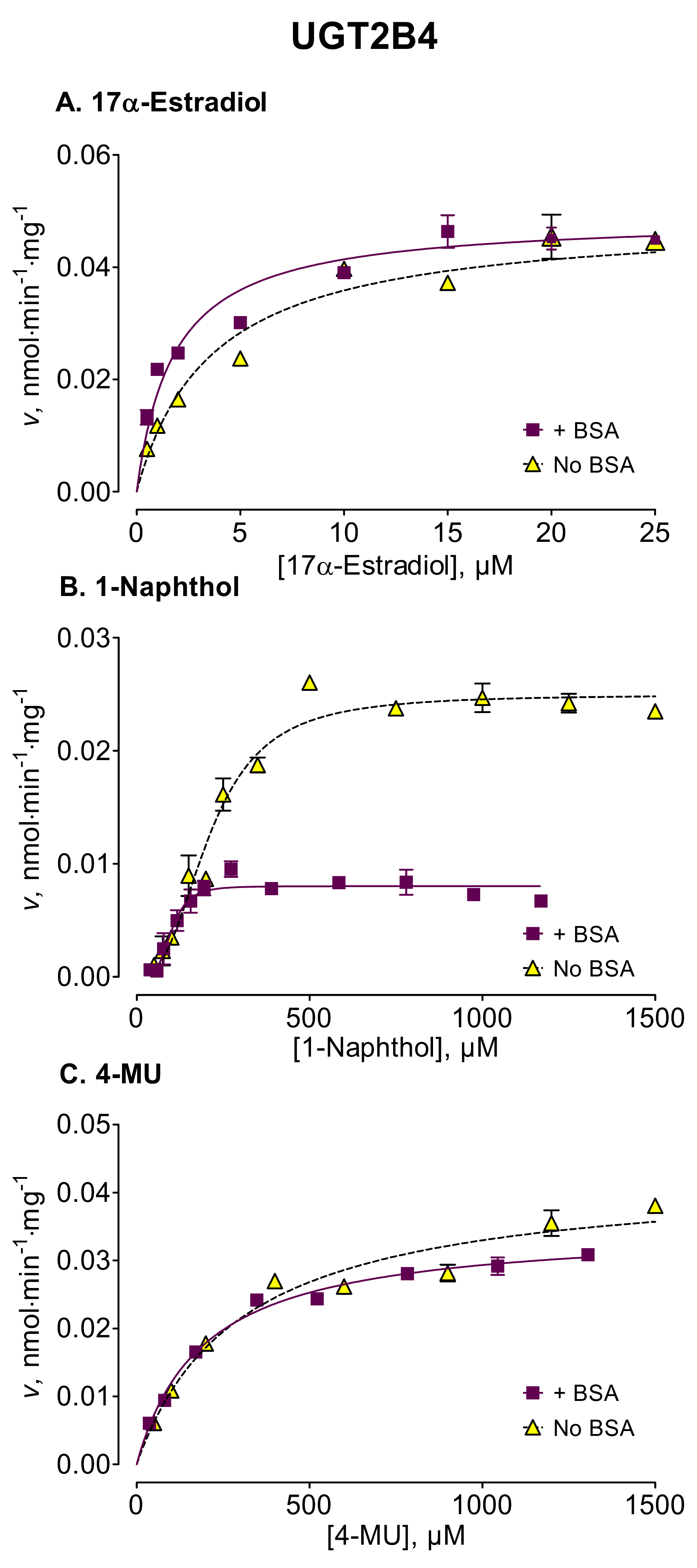

Supplement: Figure S3 — Enzyme kinetics of UGT2B4-catalyzed glucuronidation of 17α-estradiol (A), 1-naphthol (B), and 4-MU (C), in the absence and presence of BSA. The reaction with 17α-estradiol was analyzed for the formation of 17α-estradiol-17-β-D-glucuronide. The glucuronidation rates are presented as the average value ± S.E., and are expression level-normalized values. The concentrations of substrates were corrected for binding to 0.1% BSA. The determined enzyme kinetic parameters are presented in Table 3. See Materials and Methods for all further details. (TIF) [file pone.0054767.s003.tif]

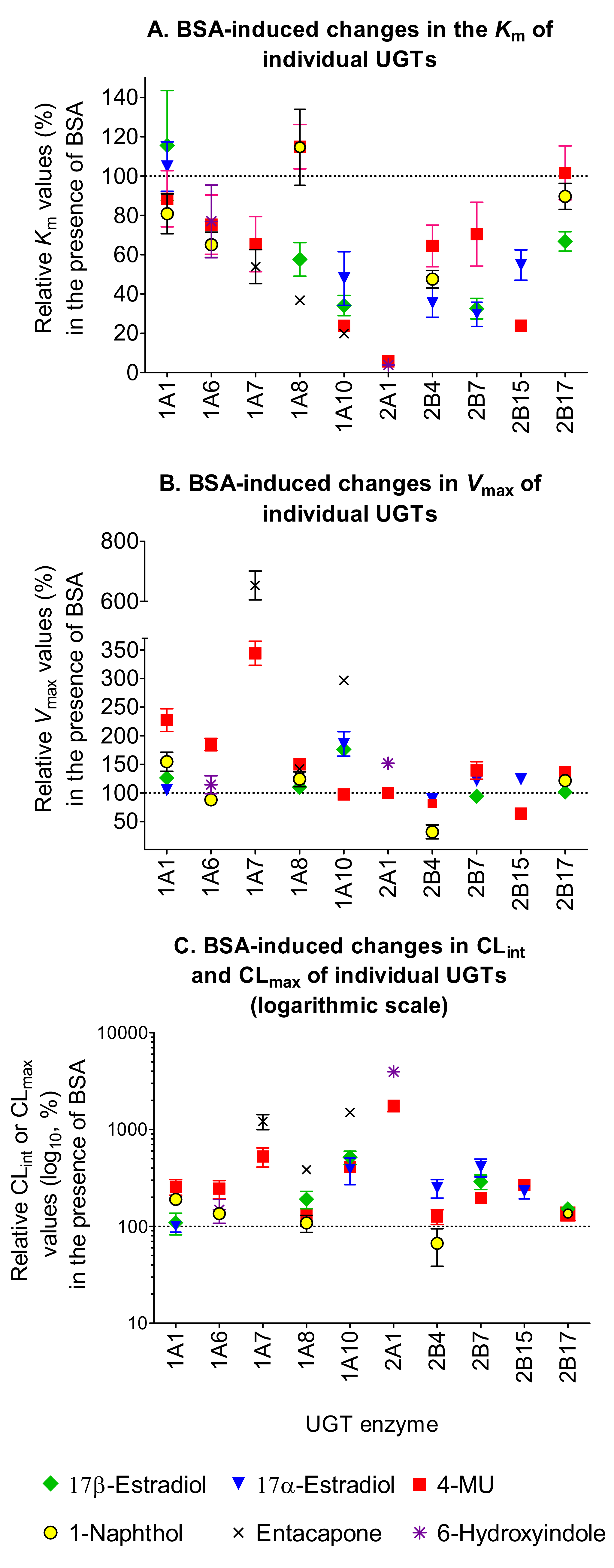

Supplement: Figure S4 — The combined effects of BSA of the K m (A), V max (B), and CLint or CLmax (C) of the ten tested human UGTs. The average values of the enzyme kinetic parameters, determined in the absence of BSA, where arbitrary assigned to 100%, and the average corresponding values of K m, V max, and CLint or CLmax in the presence of BSA were compared to the values in the absence of BSA and plotted for all the ten tested UGT enzymes. The presented errors are propagated S.E. values that take into account the errors in the parameters determined in both the absence and presence of BSA (See Materials and Methods for further details). Due to large increases of CLint or CLmax in the presence of BSA, the values of y-axis in panel C are presented on a log10 scale. (TIF) [file pone.0054767.s004.tif]

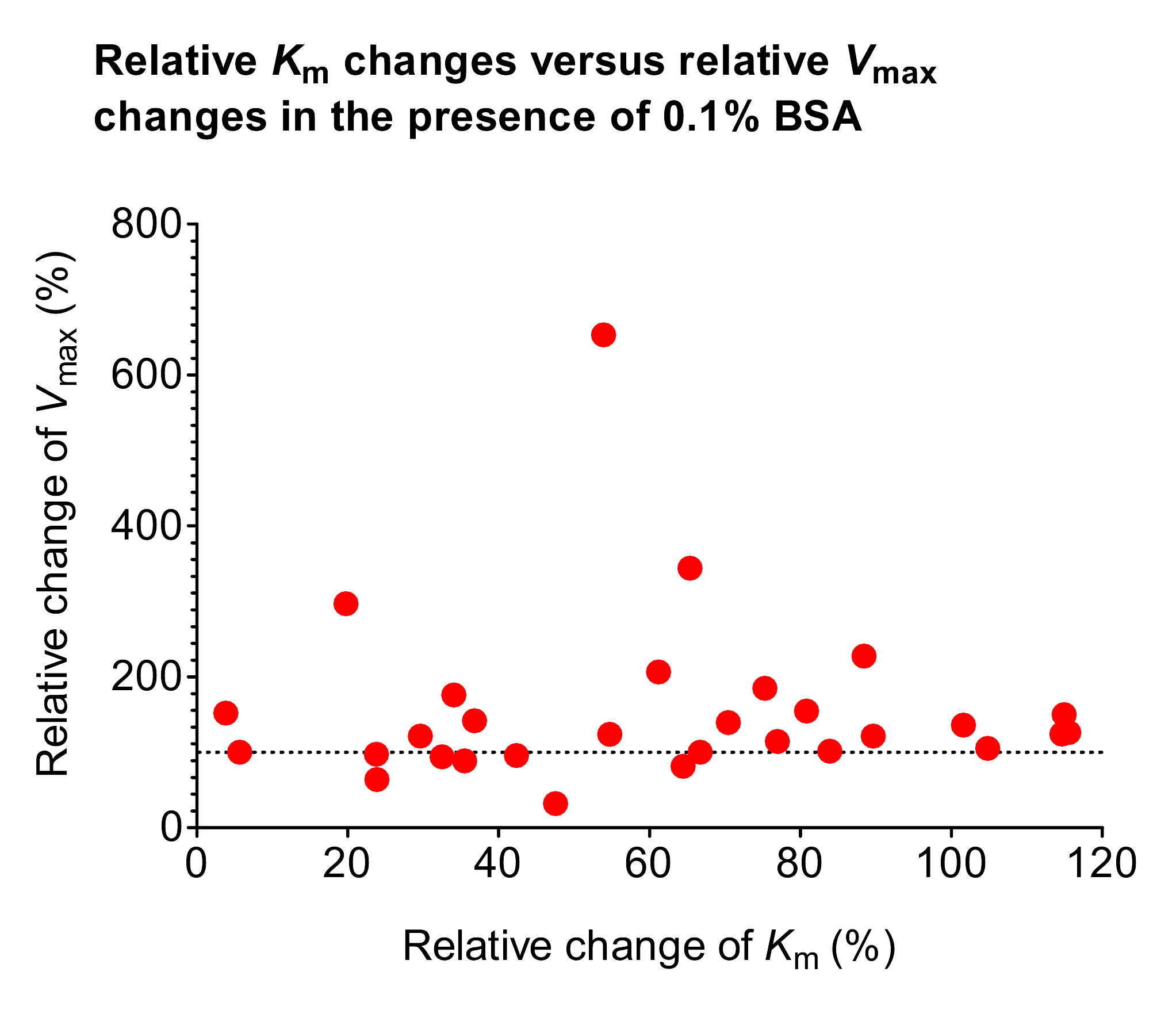

Supplement: Figure S5 — The correlation between relative K m and V max values in the presence of 0.1% BSA. The average values of enzyme kinetic parameters, determined in the absence of BSA, where arbitrary assigned to 100%, and the average relative values of K m and V max in the presence of 0.1% BSA were plotted against each other. (TIF) [file pone.0054767.s005.tif]

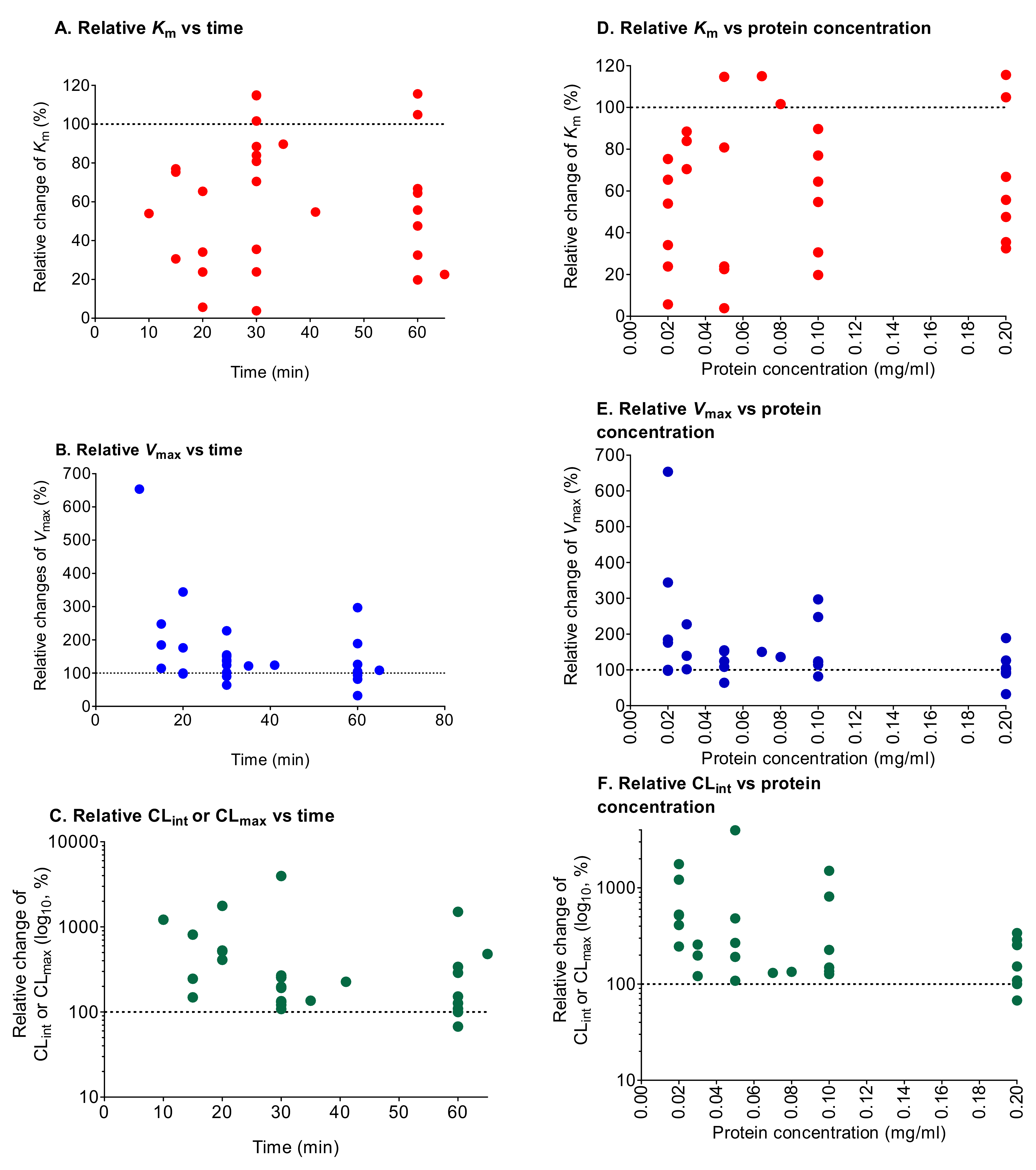

Supplement: Figure S6 — The correlation between relative enzyme kinetic parameters in the presence of 0.1% BSA ( K m, V max, and CLint or CLmax) and experimental conditions of enzyme kinetic assays, namely incubation time (panels A–C) and total protein concentration (panels D–E). The average values of enzyme kinetic parameters, determined in the absence of BSA, were arbitrary assigned to 100%, and the average relative values of K m, V max, and CLint or CLmax in the presence of 0.1% BSA were plotted against experimental conditions of the corresponding enzyme kinetic assay. For this analysis, we combined in a single graph panel the average relative values of enzyme kinetic parameters (in the presence of BSA) from all ten UGT enzymes and six substrates. Due to large increases of CLint or CLmax in the presence of 0.1% BSA, the values of y-axis are presented on log10 scale. The incubation time varied from 10 to 60 min (panels A–C), and UGT protein concentration, referring to total protein concentration in the membrane, varied from 0.02 to 0.2 mg/mL. See Materials and Methods for all further details. (TIF) [file pone.0054767.s006.tif]
